# A boundary migration model for imaging within volumetric scattering media

**DOI:** 10.1038/s41467-022-30948-7

**Published:** 2022-06-09

**Authors:** Dongyu Du, Xin Jin, Rujia Deng, Jinshi Kang, Hongkun Cao, Yihui Fan, Zhiheng Li, Haoqian Wang, Xiangyang Ji, Jingyan Song

**Affiliations:** 1 Shenzhen International Graduate School, Tsinghua University, 518055 Shenzhen, China; 2 Department of Automation, Tsinghua University, 100084 Beijing, China; 3 Institute for Brain and Cognitive Sciences, Tsinghua University, 100084 Beijing, China; 4 Beijing National Research Center for Information Science and Technology, Tsinghua University, 100084 Beijing, China; 5 Tsinghua Innovation Center in Zhuhai, 519080 Zhuhai, China

**Keywords:** Imaging and sensing, Imaging techniques

## Abstract

Effectively imaging within volumetric scattering media is of great importance and challenging especially in macroscopic applications. Recent works have demonstrated the ability to image through scattering media or within the weak volumetric scattering media using spatial distribution or temporal characteristics of the scattered field. Here, we focus on imaging Lambertian objects embedded in highly scattering media, where signal photons are dramatically attenuated during propagation and highly coupled with background photons. We address these challenges by providing a time-to-space boundary migration model (BMM) of the scattered field to convert the scattered measurements in spectral form to the scene information in the temporal domain using all of the optical signals. The experiments are conducted under two typical scattering scenarios: 2D and 3D Lambertian objects embedded in the polyethylene foam and the fog, which demonstrate the effectiveness of the proposed algorithm. It outperforms related works including time gating in terms of reconstruction precision and scattering strength. Even though the proportion of signal photons is only 0.75%, Lambertian objects located at more than 25 transport mean free paths (TMFPs), corresponding to the round-trip scattering length of more than 50 TMFPs, can be reconstructed. Also, the proposed method provides low reconstruction complexity and millisecond-scale runtime, which significantly benefits its application.

## Abstract (web summary)

Imaging in scattering media is challenging due to signal attenuation and strong coupling of scattered and signal photons. The authors present a boundary migration model of the scattered field, converting scattered measurements in spectral form to scene information in temporal domain, and image Lambertian objects in highly scattering media.

## Introduction

The ability to image within the volumetric scattering media is of great significance for a variety of macroscopic applications. It is crucial for transportation systems such as self-driving cars, airplanes, drones, and trains to operate in fog and dust^1–4^. It is highly desired for underwater robots to sense their surrounding environment in muddy water^5,6^. And it shows high potential in being applied to industrial monitoring, non-destructive testing through packaging, and collision avoidance.

**Fig. 1:**
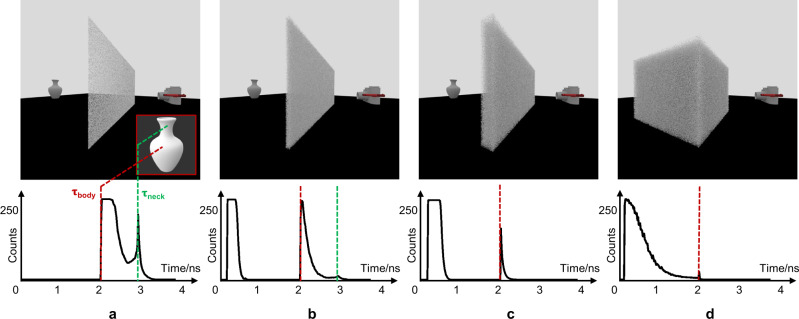
Different scattering scenarios. Different columns show different scattering scenarios: imaging through scattering media with the increase of the scattering strength and the scattering region from (**a**–**c**) and imaging within the scattering media (**d**). The bottom row shows the synthetic temporal response under different scattering scenarios (synthetic data generation is described in Methods). According to Fermat’s Principle^7^, the red and green discontinuities in the transient measurements indicate that the vase surface shape is composed of a convex body and a saddle neck (details in Supplementary Note 7).

Unfortunately, imaging within such volumetric scattering scenarios is challenging since the ballistic photons are almost submerged and the target information is all carried by the scattered photons. Using optical reflection imaging, the most general case, as an instance, light reflected by the boundaries of the hidden objects behind an ideal single-scattering layer carries the shape information^7^, like the vase surface shape in Fig. 1a, in the discontinuities of the transient measurements. With the increase in the scattering strength and the scattering region like that in Fig. 1b, c, the number of surviving target photons reaching the sensor decreases quickly. It results in a drastic increase in the number of background photons that are directly reflected by the scattering media (the left-most peak in the curves in Fig. 1b, c), and an obvious decrease in the signal photons that are reflected by the target and transmit through the scattering media (the right peaks in Fig. 1b, c). Although quantifying the disturbance^8–10^ or utilizing spatial correlation in scattering properties^11–15^ can partially recover the target behind the scattering media, they become ineffective as the target is deeply embedded in the volumetric scattering media like that in Fig. 1d, where both the light reflected by the target and the media are highly coupled and attenuated during volumetric scattering. It results in little or no direct information of the target being measured, which corresponds to a near vanished signal photon peak in Fig. 1d. The scenario becomes challenging when the surface of the embedded target is Lambertian, like the vase in Fig. 1, where the photons reflected from the target are scattered isotropically resulting in great difficulties in decoupling the photons returned by the object and the scattering media.

To alleviate limitations in imaging through or within the scattering media, some methods explore the spatial distribution of scattered photons while others analyze the temporal characteristics of scattered photons. The spatial distribution of scattered photons can be characterized by speckle correlations^11–15^ or transmission /reflectance models^1,16,17^. The former is based on the angular correlations of scattered light which is known as memory effect^18^. These methods can provide diffraction-limited passive imaging while suffering from a limited field of view and the correlation feature is no longer maintained in macroscopic volumetric scattering scenarios. Transmission/reflectance modeling-based image restoration describes the spatial distribution of scattered photons in the color space. Berman et al.^1^ and He et al.^19^ model a scattered image as a combination of the airlight and the object radiance going through a transmission matrix. The transmission coefficients are estimated by the scattered-color-formed haze-lines^1,16,17^ or the color channel with the lowest intensity, the so-called dark channel^19–21^. While they may fail in the case that the airlight is much brighter than the target radiance when the target is deeply embedded in the strong scattering media. To further separate the illumination from the object radiance, Retinex theory^22–24^ is exploited to estimate the object reflectance using the smoothness prior to lighting. However, this key assumption fails due to the multi-path effects in highly volumetric scattering media.

Some methods try to recover the object by extracting ballistic photons according to different temporal characteristics between ballistic photons and scattered photons or modeling and inverting the transmission of scattered photons. Isolating the ballistic photons reflected by the object can be achieved by time-resolved filtering^25–28^ fitting and distinguishing the time profiles of ballistic photons (Gaussian Distribution) and scattered photons (Gamma Distribution)^4^, and analyzing cross-correlation between measurements and instrumental responses^29–31^. While their effectiveness drops drastically when the number of ballistic photons approaches zero, like that shown in Fig. 1d, and the ballistic photons are highly coupled with the background photons. Different from the above methods relying on ballistic photons, inverse transmission modeling methods try to describe the propagation of scattered photons theoretically using the radiative transfer equation (RTE) and its first-order spherical harmonic approximation, the diffusion equation (DE). Diffuse optical tomography (DOT) algorithms^32–34^ calculate the analytical solution of the RTE or DE under specific initial conditions or obtain their numerical approximation. Since the inverse problems are inherently ill-posed and highly undetermined, these methods suffer from low resolution due to the computational approximation. Lyons et al.^35^ build a regularized least-square optimization model based on the diffusion equation and model the propagation of the light as a two-step operation: the illumination plane to the object and the object to the observation plane, so that it is limited to recover the 2D object and requires the priors including the object depth and the thicknesses of the two scattering layers. Additionally, the heavy computation workload for solving optimization problems results in relatively low reconstruction quality. Lindell et al.^36^ treat the analytic solution of the diffusion equation for the slab geometry (see Fig. 1b) as the blur kernel acting on the free-space propagation of light to represent the diffusion process. However, it relies on the target information carried by free-space propagation, which is unavailable in the volumetric scattering.

In this paper, we address the scattering reconstruction problem by providing a boundary migration model (BMM) of the scattered field to map the scattered electromagnetic radiation change in the volumetric scattering media to a time-to-space scattered field transformation. We prove that scattered measurements and the object information correspond to the spatial and temporal boundaries of the scattered field, respectively, based on which the space-time mapping in the frequency domain is established to convert the scattered measurements in spectral form to the scene information in the temporal domain. It alleviates the limitations caused by inverting the transmission model using incomplete information, thus producing better-quality reconstructions. The proposed algorithm performs well in regimes where the signatures of the targets are too weak to be identified from visual inspection of the measurements. We demonstrate that the proposed method outperforms related methods, including time gating, in terms of reconstruction precision and scattering strength. Lambertian objects located at 25.4 TMFPs can be reconstructed, i.e., the round-trip scattering length is 50.8 TMFPs, as the proportion of signal photons is only 0.75%. Besides, the proposed method has low reconstruction complexity, which consumes only millisecond-scale runtime.

## Results

### Boundary migration model of the scattered field

**Fig. 2:**
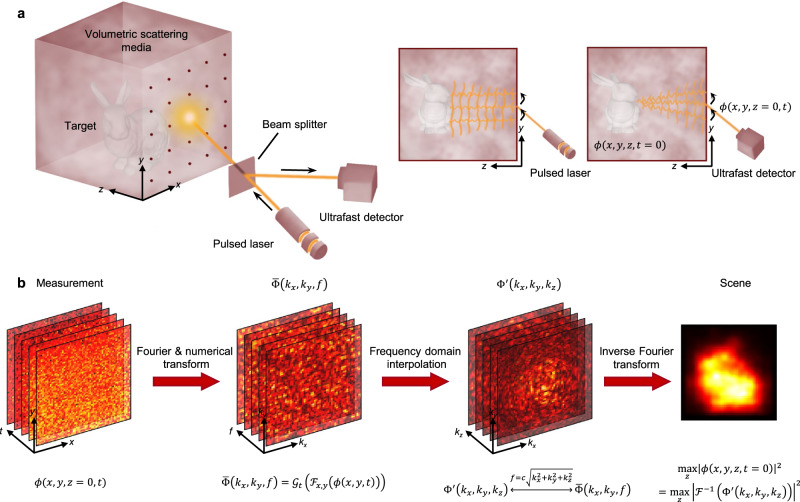
Illustration of the proposed boundary migration model of the scattered field. **a** The setup includes a pulsed laser, an ultrafast detector, a beam splitter, volumetric scattering media, and the target. Laser and detector are used to probe the time-resolved light propagation on the front surface of the scattering media. Since they share the same optical path, the light propagation in scattering media can be regarded as one-way propagation. Furthermore, supposing the target emits electromagnetic radiation at $t=0$, the scene information is encoded in the temporal boundary characteristics of the scattered field $\phi (x,y,z,t=0)$. Fixing the scanning surface at $z=0,$ the measurements are encoded in the spatial boundary characteristics of the scattered field $\phi (x,y,z=0,t)$. **b** The entire reconstruction pipeline. Taking the measurement as the input, the proposed method follows three steps to recover the scene: Fourier and numerical transform of the measurement to get $\bar{\Phi }\left({k}_{x},{k}_{y},f\right)$, time-to-space interpolation of $\bar{\Phi }\left({k}_{x},{k}_{y},f\right)$ in frequency domain to obtain ${\Phi }^{{\prime} }\left({k}_{x},{k}_{y},{k}_{z}\right)$ and inverse Fourier transform (IFT) of ${\Phi }^{{\prime} }\left({k}_{x},{k}_{y},{k}_{z}\right)$ to recover the scene information.

The boundary migration model of the scattered field is built based on the confocal time-of-flight imaging system^37–40^ as illustrated in Fig. 2a. Light emitted from an ultrafast pulsed laser illuminates a point on the surface of the volumetric scattering media, diffuses throughout the volumetric space and is back-reflected by the hidden target and scattering particles. The scattered photons are time-tagged by the ultrafast detector. Changing the illuminating points by laser scanning, a series of measurements is collected.

We parameterize the light propagation in the scattering media as a scattered field $\phi (x,y,z,t)$ in the 3D space $x,y,z,$ and time $t$. Without loss of generality, the scanning surface of the scattering media is set to be $z=0$ after calibrating the distance among the laser, the detector, and the scanning surface. Thus, the measurements captured by the detector can represent the spatial boundary characteristics of the scattered field $\phi (x,y,z=0,t)$. Since the photons are measured at the point same with illumination using confocal configuration, their propagation in the scattering media can be roughly traced back to a patch on the hidden target where the diffused light illuminates and the scattered photons originate at *t* = 0. Dividing the Cartesian space that the hidden target locates into voxels and supposing each voxel emits electromagnetic radiation, the target information is encoded in the scattered field at $\phi (x,y,z,t=0)$, which corresponds to the temporal boundary characteristics of the scattered field. Thus, we formulate imaging the objects embedded in the volumetric scattering media as a boundary migration problem that estimates $\phi (x,y,z,t=0)$ from the measurements $\phi (x,y,z=0,t)$.

A time-to-space transformation is designed in the frequency domain to migrate the spatial boundary characteristics $\phi (x,y,z=0,t)$ to the temporal boundary characteristics $\phi (x,y,z,t=0)$. The entire pipeline is shown in Fig. 2b. Modeling the light propagation inside the volumetric scattering media using the diffusion approximation^35^, the scattered field $\phi (x,y,z,t)$ can be expressed as the solution of the time-resolved diffusion equation:

1 $$\begin{array}{c}\frac{1}{c}\frac{\partial \phi (x,y,z,t)}{\partial t}-\nabla [D(x,y,z)\nabla \phi (x,y,z,t)]\\ +{\mu }_{a}(x,y,z)\phi (x,y,z,t)=s(x,y,z,t),\end{array}$$

where $s\left(x,y,z,t\right)$ is a photon source, and $c$ is the speed of light in the media. $D(x,y,z)$ is the diffusion coefficient^26^ defined by the absorption coefficient ${\mu }_{a}$ and the reduced scattering coefficient ${\mu }_{s}^{{\prime} }$. Assuming it is independent of spatial position and time, the diffusion coefficient is given by $D={\left[3\left({\mu }_{a}+{\mu }_{s}^{{\prime} }\right)\right]}^{-1}$. Differential operator $\nabla =\frac{\partial }{\partial x}+\frac{\partial }{\partial y}+\frac{\partial }{\partial z}$ is defined over the spatial coordinate. The spectrum form of Eq. (1) with respect to spatial coordinates can be described as:

2 $$\begin{array}{c}\frac{1}{c}\frac{\partial \varPhi ({k}_{x},{k}_{y},{k}_{z},t)}{\partial t}+D({k}_{x}^{2}+{k}_{y}^{2}+{k}_{z}^{2})\varPhi ({k}_{x},{k}_{y},{k}_{z},t)\\ +{\mu }_{a}\varPhi ({k}_{x},{k}_{y},{k}_{z},t)=S({k}_{x},{k}_{y},{k}_{z},t),\end{array}$$

where $S({k}_{x},{k}_{y},{k}_{z},t)$ and $\Phi ({k}_{x},{k}_{y},{k}_{z},t)$are the Fourier domain representation of $s\left(x,y,z,t\right)$ and $\phi \left(x,y,z,t\right)$ performed on the spatial dimensions, respectively; and $({k}_{x},{k}_{y},{k}_{z})$ is the wave vector indicating the direction of light propagation. Equation (2) is a first-order partial differential equation with respect to time $t$ for a fixed $({k}_{x},{k}_{y},{k}_{z})$, and its general solution has the form:

3 $$\begin{array}{c}\varPhi ({k}_{x},{k}_{y},{k}_{z},t)={{{{{{\rm{e}}}}}}}^{-c[D({k}_{x}^{2}+{k}_{y}^{2}+{k}_{z}^{2})+{\mu }_{a}]t}\left[C({k}_{x},{k}_{y},{k}_{z})\right.\\ \left. +\int cS({k}_{x},{k}_{y},{k}_{z},t){{{{{{\rm{e}}}}}}}^{c[D({k}_{x}^{2}+{k}_{y}^{2}+{k}_{z}^{2})+{\mu }_{a}]t}{{{{{\rm{d}}}}}}t\right],\end{array}$$

where $C({k}_{x},{k}_{y},{k}_{z})$ is introduced by the homogeneous solution of Eq. (2) and is an arbitrary function independent of time. Equation (3) gives the specific expression of the spectrum of $\phi \left(x,y,z,t\right)$ on the spatial dimensions, based on which $\phi \left(x,y,z,t\right)$ can be derived as:

4 $$\begin{array}{c}\phi (x,y,z,t)=\frac{1}{{(2\pi )}^{3}}\\ \cdot \iiint \underbrace{\bigg(C({k}_{x},{k}_{y},{k}_{z})+\int cS({k}_{x},{k}_{y},{k}_{z},t){{{{{{\rm{e}}}}}}}^{c[D({k}_{x}^{2}+{k}_{y}^{2}+{k}_{z}^{2})+{\mu }_{a}]t}{{{{{\rm{d}}}}}}t\bigg)}_{={\varPhi}^ {\prime} ({k}_{x},{k}_{y},{k}_{z})}\\ \cdot {{{{{{\rm{e}}}}}}}^{-c[D({k}_{x}^{2}+{k}_{y}^{2}+{k}_{z}^{2})+{\mu }_{a}]t+i({k}_{x}x+{k}_{y}y+{k}_{z}z)}{{{{{\rm{d}}}}}}{k}_{x}{{{{{\rm{d}}}}}}{k}_{y}{{{{{\rm{d}}}}}}{k}_{z}.\end{array}$$

Since the target information is the temporal boundary characteristics of $\phi \left(x,y,z,t\right)$, i.e., $t=0$, the exponential part of Eq. (4) can be simplified to express the Fourier transformation relationship between $\phi \left(x,y,z,t=0\right)$ and its spectrum ${\Phi }^{{\prime} }\left({k}_{x},{k}_{y},{k}_{z}\right)$. Correspondingly, the measurement is described as the spatial boundary characteristics of $\phi \left(x,y,z,t\right)$, i.e., $z=0$. However, directly setting $z=0$ in Eq. (4) results in a complicated form, making it computationally impossible to establish the transformation relationship between $\phi \left(x,y,z,t=0\right)$ and $\phi \left(x,y,z=0,t\right)$. Inspecting the exponential component in Eq. (4), the square of the wave vector module, ${k}_{x}^{2}+{k}_{y}^{2}+{k}_{z}^{2}$, can be related to the optical wavelength by ${{{{{\rm{|}}}}}}({k}_{x},{k}_{y},{k}_{z}){{{{{\rm{|}}}}}}=1/\lambda$ and the wavelength equals the frequency of the light by $\lambda =c/f$. Thus, the wave vector can be transformed to the frequency by $f=c\sqrt{{k}_{x}^{2}+{k}_{y}^{2}+{k}_{z}^{2}}$, which is referred to as the dispersion relation for scalar wave. Assuming that light propagates towards $+{k}_{z}$ (the direction of scattering media), the wavenumber ${k}_{z}$ can be defined as ${k}_{z}=\sqrt{{f}^{2}/{c}^{2}-{k}_{x}^{2}-{k}_{y}^{2}}.$ According to the rule of calculus, a variable change can be performed in Eq. (4) using the dispersion relation of ${k}_{z}$ and its Jacobian. Then, Eq. (4) can be described as an integral over ${k}_{x},{k}_{y}$, and $f$, which is given by

5 $$\begin{array}{c}\phi (x,y,z,t)=\frac{1}{{(2\pi )}{}^{3}}\\ \cdot \iiint \underbrace{\left(\begin{array}{c}\frac{|f|/{c}^{2}}{\sqrt{{f}^{2}/{c}^{2}-{k}_{x}^{2}-{k}_{y}^{2}}}\left[C\left({k}_{x},{k}_{y},\sqrt{{f}^{2}/{c}^{2}-{k}_{x}^{2}-{k}_{y}^{2}}\right)\right.\\ \left. +\int cS\left({k}_{x},{k}_{y},\sqrt{{f}^{2}/{c}^{2}-{k}_{x}^{2}-{k}_{y}^{2}},t\right){{{{{{\rm{e}}}}}}}^{[D\frac{{f}^{2}}{{c}^{2}}+c{\mu }_{a}]t}{{{{{\rm{d}}}}}}t\right] \end{array}\right)}_{\begin{array}{c}=\bar{\varPhi }({k}_{x},{k}_{y},f)\\ \cdot {{{{{{\rm{e}}}}}}}^{-\left[D\frac{{f}^{2}}{{c}^{2}}+c{\mu }_{a}\right]t+i\left({k}_{x}x+{k}_{y}y+z\sqrt{{(f/c)}{}^{2}-{k}_{x}^{2}-{k}_{y}^{2}}\right)}{{{{{\rm{d}}}}}}{k}_{x}{{{{{\rm{d}}}}}}{k}_{y}{{{{{\rm{d}}}}}}f.\end{array}}\end{array}$$

Equation (5) gives the complete reformulation of the scattered field $\phi (x,y,z,t)$ in $\left({k}_{x},{k}_{y},f\right)$ domain. Setting $z=0$ in Eq. (5) gives a simplified spatial boundary condition of the scattered field, i.e., the measurement $\phi \left(x,y,z=0,t\right)$, by

6 $$\begin{array}{c}\phi (x,y,z=0,t)=\frac{1}{{(2\pi )}^{3}}\iiint \overline{\varPhi }({k}_{x},{k}_{y},f)\\ {{{{{{\rm{e}}}}}}}^{-[D\frac{{f}^{2}}{c}+c{\mu }_{a}]t+i({k}_{x}x+{k}_{y}y)}{{{{{\rm{d}}}}}}{k}_{x}{{{{{\rm{d}}}}}}{k}_{y}{{{{{\rm{d}}}}}}f.\end{array}$$

As shown in Eq. (6), $\phi \left(x,y,z=0,t\right)$ is derived from $\bar{\Phi }({k}_{x},{k}_{y},f)$ by inverse Fourier transform (IFT) over ${k}_{x},{k}_{y}$ and numerical transform over $f$ (details are described in Methods). Then the relationship between the spatial and temporal boundary conditions of the BMM model can be established in the frequency domain by ${\Phi }^{{\prime} }\left({k}_{x},{k}_{y},{k}_{z}\right)$ and $\bar{\Phi }\left({k}_{x},{k}_{y},f\right).$ Equations (4 and 5) show that the transformation between ${\Phi }^{{\prime} }$ and $\bar{\Phi }$ contains a weight coefficient and 1D interpolation. The interpolation method in the frequency domain, also known as frequency-wavenumber (*f*-*k*) migration, is proposed by Stolt in seismic imaging^38,41^ and recently has been applied in a wide range of areas including synthetic aperture radar^42^, ultrasound imaging^43^, optical imaging^40^ and so on.

Summarizing the process of recovering $\phi \left(x,y,z,t=0\right)$ from $\phi \left(x,y,z=0,t\right)$, BMM model follows: 2D Fourier transform incorporating the 1D numerical transform of the $\phi \left(x,y,z=0,t\right)$ using Eq. (6) to obtain $\bar{\Phi }\left({k}_{x},{k}_{y},f\right)$; time-to-space interpolation in frequency domain using the dispersion relation of $f$ and its Jacobian, taking similar steps as Eq. (5) to get ${\Phi }^{{\prime} }({k}_{x},{k}_{y},{k}_{z})$; 3D inverse Fourier transform of ${\Phi }^{{\prime} }({k}_{x},{k}_{y},{k}_{z})$ to obtain $\phi \left(x,y,z,t=0\right)$ using Eq. (4).

BMM is computationally bounded by 2D Fourier transform and 1D numerical transform in Eq. (6). Defining $N$ as the number of pixels along each dimension of the measurement volume, the computational complexity of the proposed method is $O({N}^{3}{{\log }}N)$.

**Fig. 3:**
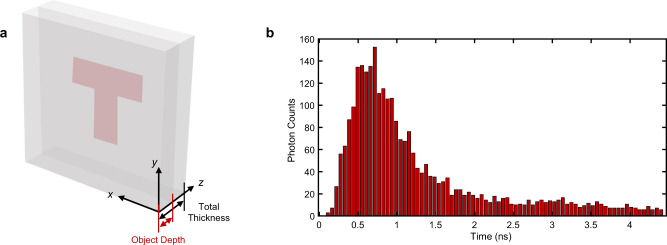
Scenario layout and the temporal response in the polyethylene foam. **a** The scenario layout of the target and the polyethylene foam. Taking the target letter ‘T’ as an example, it is located at 8 cm in the foam and the total thickness of the foam is 15 cm. The object depth is defined as the distance between the object and the front surface of the media. **b** The temporal response at one of the scanning points is captured by the ultrafast detector in the scenario shown in (**a**). Since the foam is an isotropic scattering medium where photon scatters at all angles with equal probability, the signal photons reflected by the target are highly coupled with background photons reflected by the scattering media.

**Fig. 4:**
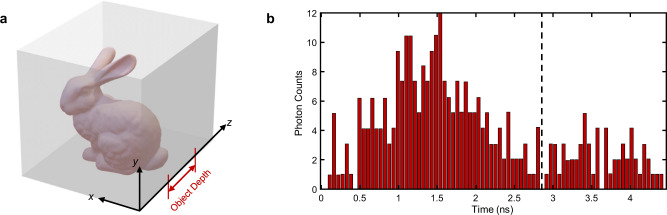
Scenario layout and the temporal response in the fog. **a** The scenario layout of the target and the fog chamber. Taking the target ‘Bunny’ as an example, its depth range is 40–60 cm and the total depth of the fog chamber is 150 cm. The object depth range is defined as the distance range between the object and the front surface of the media. **b** The temporal response at one of the scanning points is captured by the ultrafast detector in the scenario shown in (**a**). Affected by the time variability of the fog, the temporal response is disturbed by the dynamic noise. Since the backscattered light has little effect on signal photons in the fog, the photons reflected by the object and those do not reach the object can be distinguished in the temporal response taking depth as a prior as the dashed line divided.

We evaluate the effectiveness of our algorithm in two typical scattering scenarios: 2D and 3D Lambertian objects embedded in the polyethylene foam and the fog. The scenario layouts are shown in Figs. 3a and 4a. The strength of scattering, absorption, and propagation angle of the two scattering media are quite different. The polyethylene foam is a homogeneous static isotropic scattering medium while the fog is an inhomogeneous dynamic forward scattering medium^44–46^. They produce different temporal responses as shown in Figs. 3b and 4b respectively. The former is affected by backscattered light in which case the signal photons are highly coupled with the background photons. The latter is affected by the time variability of the fog so that the temporal distribution of signal photons is superimposed with dynamic noise. Both cases exacerbate the reconstruction difficulty while we demonstrate the capability of our algorithm in these two volumetric scattering scenarios.

### Reconstruction of Lambertian objects in the polyethylene foam

**Fig. 5:**
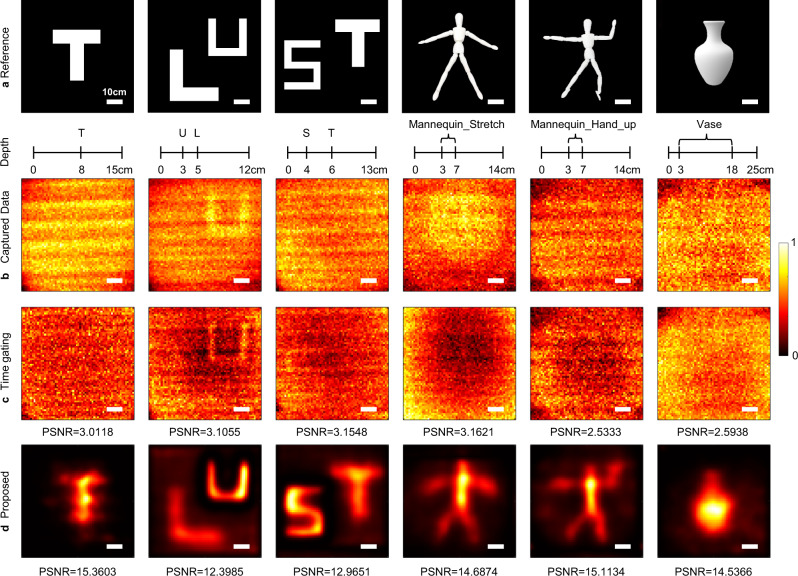
Comparisons on reconstructing Lambertian objects in the polyethylene foam. **a** References of 2D, multi-depth, and 3D Lambertian objects used in this experiment. The total thicknesses of polyethylene foam and object depths are marked in the scale below each picture. Taking the letter ‘T’ in the first column as an example, the total thickness of the foam is 15 cm and the ‘T’ is located at 8 cm. All scale bars indicate 10 cm. **b** Images directly captured by the detector. **c** Time-gated results which are taken by combining multiple time bins corresponding to the object depths. **d** Reconstructions using the proposed method. The peak signal-to-ratio (PSNR) is calculated as the quantitative indicator to compare time gating and the proposed algorithm using the references in (**a**). The results are marked in (**c**, **d**). PSNR is in dB.

Figure 5 presents the reconstruction results of the BMM model for recovering 2D, multi-depth, and 3D Lambertian objects embedded in the polyethylene foam. The time-of-flight (ToF) measurements are captured by the experimental setup described in detail in Methods and Supplementary Note 1. The imaging scenario is given in Supplementary Fig. 2. The characteristics of the polyethylene foam are estimated as: the reduced scattering coefficient ${\mu }_{s}^{{\prime} }$ is $3.1377\pm 0.35{{{{{{\rm{cm}}}}}}}^{-1}$ and the absorption coefficient ${\mu }_{a}$ is $3.3348\times {10}^{-2}\pm 2.6\times {10}^{-3}{{{{{{\rm{cm}}}}}}}^{-1}$(details in Supplementary Note 2). In this case, the length of one TMFP is given by ${l}^{* }=\frac{1}{{\mu }_{s}^{{\prime} }}\approx 0.3187\pm 0.0356{{{{{\rm{cm}}}}}}$, which represents the distance over which photons lose their knowledge of initial propagation direction. Since the total thicknesses of polyethylene foam used in this experiment, ranging from 12 cm to 25 cm, are far larger than one TMFP, the signal photons are almost submerged. Besides, the foam is an isotropic scattering medium where photons scatter in all angles with equal probability so that signal photons are highly coupled with backscattered photons, as illustrated by the temporal response curve in Fig. 3b. The test objects include a single letter ‘T’, two-depth letters ‘LU’, ‘ST’ and 3D objects ‘Mannequin_Stretch’, ‘Mannequin_Hand_up’, ‘Vase’ as shown in Fig. 5. The total thickness of the foam and object depth are marked in the scale below each picture. The scenario layout of the target and the polyethylene foam is shown in Fig. 3a. The object depth is defined as the distance between the object and the front surface of scattering media. The material information of the targets is detailed in Methods. All measurements shown in Fig. 5b are captured by scanning a 45 × 45 cm area, with sampling points spaced by $0.7031{{{{{\rm{cm}}}}}}$ in the *x* and *y* direction so that the spatial pixel resolution is 64 × 64. The temporal resolution of the measurement is $55{{{{{\rm{ps}}}}}}$, which is determined by the ultrafast detector.

Figure 5 presents the images directly captured by the detector and reconstructed by time gating and the proposed method. For a single plane object, the time-gated results are taken by selecting several time bins around the depth of the object. For objects with multiple discrete depths or continuous depth, time-gated results are taken by combining multiple time-shifted gates corresponding to different object depths^47^. Since it is challenging to decouple the photons reflected by the object and those reflected by the scattering media in this scenario, the signatures of the objects cannot be distinguished from time-gated reconstructions shown in Fig. 5c. Even though time gating can recover objects at a relatively close depth, such as the letter ‘U’ in data ‘LU’, the result has a low signal-to-noise ratio. Additionally, time gating relies on the prior depth map. In contrast, benefitting from the accurate model and computationally using all of the optical signals, our method recovers all targets correctly and provides a good qualitative agreement with the ground truth, as shown in Fig. 5d. Besides, the peak signal-to-noise ratio (PSNR) is calculated as the quantitative indicator to compare time gating and the proposed method as marked in Fig. 5c, d. It can be found that the proposed method outperforms time gating in all experiments from both subjective and objective evaluation.

We note that our algorithm provides a legible result of letter ‘T’ at a depth of 8 cm, corresponding to the one-way scattering length of 25.4 TMFPs, where signal photons are significantly attenuated as shown in Supplementary Fig. 10. In this case, time gating fails to provide any helpful information regardless of the presence or absence of the object. Additional comparison results of time gating and proposed method for Lambertian letter ‘T’ at other depths are shown in Supplementary Table. 2. When there are multiple 2D objects with different discrete depths or 3D objects with continuous depth in the scenario, signal photons reflected from different depths overlap in their temporal distribution, which leads to a more challenging reconstruction task. In this case, the method based on isolating the ballistic photons such as time gating has poor performance in reconstructing the distant objects. In contrast, our method accurately models the propagation of scattered light across the whole three-dimensional space so that we can reconstruct multi-depth objects simultaneously and even 3D objects.

### Reconstruction of Lambertian objects in the fog

**Fig. 6:**
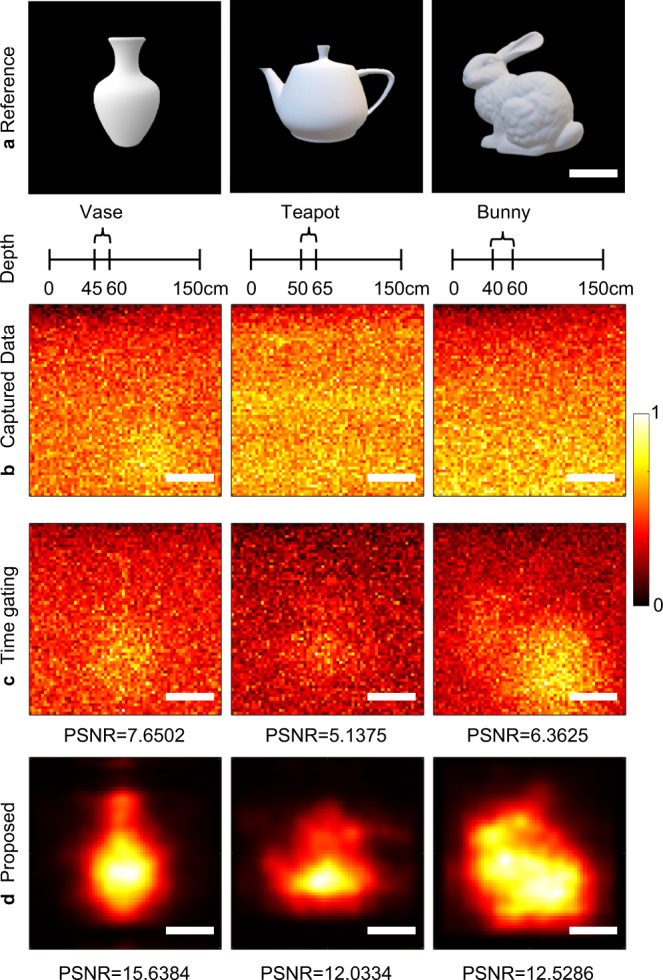
Comparisons on reconstructing Lambertian objects with complex structures in the fog. **a** References of 3D Lambertian objects used in this experiment. The total depth of the fog chamber and object depths are marked in the scale below each picture. Taking the ‘Vase’ in the first column as an example, the total depth of the fog chamber is 150 cm and ‘Vase’ is located at a depth range of 45-60 cm. All scale bars indicate 10 cm. **b** Images directly captured by the ultrafast detector; **c** Time-gated results which are taken by combining multiple time-shift bins corresponding to the object depth range. **d** Reconstructions using the proposed method. PSNR is calculated as the quantitative indicator to compare time gating and the proposed algorithm using the references in (**a**). The results are marked in (**c**, **d**). PSNR is in dB.

In this experiment, we evaluate the reconstruction performance of our algorithm in a time-varying dynamic scattering scenario. An artificial fog chamber provides the scattering environment with a size of 50 × 50 × 150 cm. Supplementary Fig. 3 gives the imaging scenario. The transport coefficient ${\mu }_{t}$ of the fog, given by the sum of scattering coefficient ${\mu }_{s}$ and absorption coefficient ${\mu }_{a}$, is estimated as $0.2403{{{{{\rm{c}}}}}}{{{{{{\rm{m}}}}}}}^{-1}$(see Supplementary Note 2 for details). Since the fog is time-varying, the temporal response is disturbed by dynamic noise as shown in Fig. 4b, which makes it challenging to reconstruct the objects. The test objects in this experiment are shown in Fig. 6a, including three 3D Lambertian targets, ‘Vase’, ‘Teapot’ and ‘Bunny’. The object depth of each target is marked below each picture. The scenario layout of the target and the fog chamber is given in Fig. 4a. As shown in Supplementary Fig. 7, the front surface of the fog chamber is sampled at 64 × 64 locations over a 25 × 25 cm region. Traveling along the direction of the light, the scanning range of the depth plane where the object is located is roughly 45 × 45 cm.

The reconstruction performance of time gating and the proposed method is compared in Fig. 6. The reconstructions in Fig. 6d, especially the details such as the handle and lid of the ‘Teapot’, and the ear of the ‘Bunny’, show the robustness of our algorithm in such a time-varying scattering scenario. Since the fog is a typical forward scattering medium where backscattered light has little effect on signal photons, compared to the case of the foam, the photons reflected by the object and those do not reach the object can be distinguished in the temporal response taking depth as a prior as shown in Fig. 4b. Therefore, the time-gated images also provide partial discernible features of objects, as shown in Fig. 6c. Using PSNR as the quantitative evaluator in Fig. 6c, d, our algorithm outperforms time gating subjectively and objectively in all experiments.

Exposure time per scan position, photon counts per histogram, and runtime for all experiments are summarized in Supplementary Table 9. To recover a volume of 64 × 64 × 128 voxels, the runtime for MATLAB is approximately 400-500 ms on a conventional CPU (2.3 GHz Intel Core i5). Compared with the real-time GPU-based methods^48^, a similar reconstruction speed can be obtained by the proposed method. The exposure time is 60 ms per scan position in the foam scenario and 20 ms in the fog scenario. Besides, the photon counts per histogram are in the order of thousands in the foam scenario and hundreds in the fog scenario. Both exposure time and photon counts used in our experiment are smaller than those in other related works^36,40^. The low requirements of data acquisition and millisecond-scale runtime of the proposed algorithm show the potential for real-time applications.

## Discussion

We provide a new insight for tackling the problem of imaging within the volumetric scattering media, which regards the scattering process as a boundary value migration of the scattered field. Unlike the methods based on ballistic photons^1,16,17,19–24^, our method focuses on the regime where the signal photons are almost submerged. Distinct from the methods that directly invert the transmission model of scattered photons under strict initial conditions^32–36^, we treat the mapping from the measurements to the scene as a time-to-space transformation of the scattered field without any computational approximation, thus providing better-quality reconstructions. Additionally, our method does not require any prior assumption and can handle challenging scattering cases such as recovering complex Lambertian objects.

We have demonstrated the ability of the proposed method in reconstructing Lambertian objects in Figs. 5 and 6. It is challenging since Lambertian surfaces diffuse the photons isotropically so that the photons returned by the object are highly coupled with those returned by the scattering media, as shown in Supplementary Fig. 10, and makes the separation of signal photons more difficult. It becomes more challenging with the increase in the scattering strength of the media. Our method also works well for the much easier case in reconstructing non-Lambertian objects, since the retroreflective surfaces on them can considerably increase the direct-reflected signal photons with a much larger difference from the background scattered ones. We show the robustness of our algorithm for reconstructing objects with different albedo surfaces in Supplementary Table 4.

Validity analysis of the proposed algorithm is discussed in Supplementary Note 3. Since the diffusion equation treats light propagation in the scattering media as a random walk^32^, photons diffused inside the scattering media follow some defined probability rules, which can be regarded as the quantitative indications to determine whether the diffusion approximation breaks down. In particular, the average time of photon arrival, described as the mean time consumed by a photon flying through the scattering media, is one of the quantitative indications^49^. It is theoretically given by $\Delta t=a\frac{{d}^{2}}{6{Dc}}$ and $a$ is a proportionality factor^50^. We analyze the linear relationship between the measured average arrival time and the theoretical value and finally verify that all the experiments are within the diffusion equation’s validity range, as shown in Supplementary Fig. 8. Additionally, we demonstrate that the validity range of our algorithm is theoretically unbounded while related to the signal-to-background ratio (SBR) of the measurements in practice, which depends on the characteristics of the physical setup. Using our experimental setup, the proposed method can reconstruct Lambertian objects embedded in the highly scattering media with one-way scattering length of 25.4 TMFPs, where the SBR of measurement is only 0.75%. Furthermore, if we increase the SBR by using retroreflective objects, the upper bound of the validity range will increase to 28.5 TMFPs, corresponding to the round-trip scattering length of 57 TMFPs. Supplementary Table 2 and 3 show the reconstructions for Lambertian objects and retroreflective objects at different depths.

The resolution of our system in the polyethylene foam scattering scenario is derived using the full width at half maximum (FWHM) criterion. In our analysis, the minimal distance of two points that can be distinguished is primarily determined by the lateral diffusion of light. The spatial distribution of the laterally diffused spot is calculated by the theoretical solution of the diffusion equation for slab geometry shown in Supplementary Eq. (1). And then the resolution of the system is derived by the FWHM of the diffused spot. We demonstrate that the resolution is an increasing function of the object depth, as shown in Supplementary Fig. 11. A resolution chart is used to test the actual resolution at different depths. Supplementary Fig. 12 indicates that the experimental results agree with the theoretical resolutions well. The theoretical resolutions of our system in polyethylene foam scattering scenario are 3.3 cm at the depth of 3 cm, 4.2 cm at the depth of 4 cm, and 5.0 cm at the depth of 5 cm.

Although the experiments shown in Figs. 5 and 6 are performed under dark conditions, we have demonstrated that our algorithm is robust to ambient illumination. The change of illumination mode corresponds to the change of the source term on the right side of Eq. (1). The source term is implicitly contained in two boundary conditions, ${\Phi }^{{\prime} }({k}_{x},{k}_{y},{k}_{z})$ and $\bar{\Phi }({k}_{x},{k}_{y},f)$ as shown in Eqs. (4) and (6). Since a boundary value migration of the scattered field is used to tackle the scattering problem rather than directly calculate the analytical solution, the specific form of the source term is not important. We evaluate the reconstruction performance under ambient illumination which is provided by several 72 W fluorescent lamps. As shown in Supplementary Fig. 13b, ambient illumination affects the detection of the photons by the sensor and results in the increase of noise. However, Supplementary Table 5 demonstrates that our algorithm performs well under ambient illumination as it does in the dark environment.

With respect to prior works in the relevant area, we compare the proposed algorithm with methods *f*-*k*^40^ and CDT^36^ which are also related to the idea of boundary value migration of the wavefield. *f*-*k* and CDT are proposed in non-line-of-sight (NLoS) imaging and seeing through the scattering media, respectively. Both imaging scenarios are shown in Supplementary Fig. 14a, b. The differences among these three methods in principle are: *f*-*k* is based on wave equation which only considers the light propagation in the free space; CDT models the scattering effect as a blur kernel acting on the free-space propagation; the proposed method accurately models the light propagation in the scattering media based on diffusion equation. The difference is also reflected in the corresponding temporal response as shown in Supplementary Fig. 14, which indicates that both *f*-*k* and CDT rely on the signal photons retained by light propagation in the free space. In contrast, our method can work in highly scattering media where the signal photons are almost submerged. We compare the performance of *f*-*k*, CDT, and our algorithm in the volumetric scattering scenario. As shown in Supplementary Table 6–8, *f*-*k* and CDT can only provide limited object information when the scattering strength is weak and completely fail in the highly scattering scenario, while our algorithm successfully recovers the objects in all experiments and provides the best performance. Additionally, our data acquisition requires less exposure time and photon counts compared with the data used in *f*-*k* and CDT described in the respective papers. And the runtime of our algorithm is almost equal to that of *f*-*k* and around 3 times faster than that of CDT as shown in Supplementary Table 9.

The limitation of our algorithm in depth reconstruction is discussed in detail in Supplementary Note 8. In the derivation described above, the depth information is included from temporal boundary condition $\phi (x,y,z,t=0)$ to its spectrum ${\Phi}^{\prime} ({k}_{x},{k}_{y},{k}_{z})$, then to the interpolated field $\bar{\Phi }({k}_{x},{k}_{y},f)$, and finally to the spatial boundary condition $\phi \left(x,y,z=0,t\right),$ i.e., the measurements. In this case, the depth information of the object is reflected in the temporal information of the measurements. However, since the transformation from $\phi (x,y,z=0,t)$ to $\bar{\Phi }({k}_{x},{k}_{y},f)$ over $t$ is an ill-conditioned problem, where the transfer matrix $A$ between $f$ and $t$ is sparse as shown in Eq. (8), solving it affects the accuracy of depth reconstruction. Fortunately, the rough depth of the object can be directly estimated from the temporal response as shown in Fig. 4 and it can be used to calibrate the original depth reconstructed by our method to recover the depth of the object. We also compare the depth reconstructions of time gating, *f-k*, CDT and our algorithm in Supplementary Table 10.

We present further directions to improve the reconstruction performance of our algorithm. Since the expected value in the $({k}_{x},{k}_{z})$ domain is not always coming from the grid nodes in $({k}_{x},f)$ domain, approximations are needed in this process, which causes some resolution loss in the reconstructions. Increasing the sampling density or using a more efficient interpolation method may alleviate this limitation. Moreover, although we have demonstrated the proposed method’s ability in dynamic scattering scenarios, adding dynamic noise distribution to the current model or modeling light propagation in inhomogeneous scattering media using the radiative transfer equation can provide better performance.

## Methods

### Details of the experimental setup

The measurements used in the experiments are captured using a hardware prototype, as shown in Supplementary Fig. 1. In this setup, a 20 fs pulsed laser (Coherent Vitara-T) and a 32 × 32 array of single-photon avalanche diode (SPAD) detector (Photon Force PF32) illuminate and image at the same point on the front surface of the scattering media, operating in the confocal configuration through a polarizing beam splitter (Thorlabs PBS255). The pulsed laser has a wavelength of 808 nm at an 80 MHz repetition rate and 580 mW average power. Each pixel of the SPAD array has its own photon counting module and operates in a time-correlated single photon counting (TCSPC) mode with a photon timing accuracy of 55 ps. The data of several spatial positions in the SPAD array are selected and averaged as the measurement of the current illumination point. The pulsed laser and SPAD array cooperate in scanning a grid of 64 × 64 points on the front surface of the media using a pair of galvanometer mirrors (Thorlabs GVS012) controlled with a data acquisition device (NI-DAQ USB-6343). The delayer unit is used to shape the synchronization signal output by the laser into a standard Transistor-Transistor Logic (TTL) signal, which is then used as the acquisition trigger signal of SPAD.

The target ‘Mannequin_Stretch’ and ‘Mannequin_Hand_up’ shown in Fig. 4 are made of pine wood painted with white acrylic. The plane targets ‘T’, ‘LU’, ‘ST’ are made of white matte papers. The targets ‘Vase’, ‘Teapot’ and ‘Bunny’ shown in Fig. 6 are 3D printed using resin (C-UV-9400E). The retroreflective objects shown in Supplementary Tables 3 and 4 are made of microprism reflective films, which are commonly used in traffic signs.

### Synthetic data generation

The temporal responses shown in Fig. 1 are simulated on a transient rendering platform MitsubaToFRenderer proposed by Pediredla et al. who developed a path-sampling technique and incorporated it within a steady-state rendering framework of Mitsuba^51,52^. In our simulation, a bidirectional path tracer integrator is used to model the light transport within the scattering media. The setup consists of a single-pixel ultrafast detector with ${\sim} 1^\circ$ field of view and a collimated beam source that radiates power along a fixed ray. The source and detector operate in the confocal configuration and cooperate to focus a point on the surface of the media to generate the temporal response. The ideal single-scattering layer shown in Fig. 1a is modeled by a smooth diffuse transmitter where any incident light is diffusely scattered from the other side. The homogeneous participating media is used to model the scattering media in Fig. 1b-d, which is characterized by the scattering coefficient ${\mu }_{s}$, the absorption coefficient ${\mu }_{a}$ and the phase function. The temporal resolution of the system is set to 32 ps. In addition, a hybrid sampler that combines the Quasi-Monte Carlo sequence^53^ is used to numerically sample the optical path with a sample count of 1024.

### Calculation of the spatial boundary conditions, ${{{{{\boldsymbol{\phi }}}}}}\left({{{{{\boldsymbol{x}}}}}},{{{{{\boldsymbol{y}}}}}},{{{{{\boldsymbol{z}}}}}}={{{{{\bf{0}}}}}},{{{{{\boldsymbol{t}}}}}}\right)$

In this section, we derive the spatial boundary condition of scattered field $\phi \left(x,y,z=0,t\right)$ from $\bar{\Phi }({k}_{x},{k}_{y},f)$ using Eq. (6). Two of the integrations in Eq. (6) over ${k}_{x}$ and ${k}_{y}$ are both inverse Fourier transforms that can be done rapidly by employing inverse fast Fourier transform (IFFT). The integration over $f$ in Eq. (6) can be expressed as:

7 $$\phi (t)=\frac{1}{2\pi }\int \overline{\varPhi }(f){{{{{{\rm{e}}}}}}}^{-[D\frac{{f}^{2}}{c}+c{\mu }_{a}]t}{{{{{\rm{d}}}}}}f.$$

The continuous scattered field $\phi$ and the operator $\bar{\Phi }$ are implemented with discrete matrix operations in practice. Therefore, rewriting Eq. (7) as the form of matrix multiplication $\phi =A\bar{\Phi }$ as:

8 $${\left[\begin{array}{c}\phi ({t}_{1})\\ \vdots \\ \phi ({t}_{M})\end{array}\right]}_{M\times 1}=\frac{1}{2\pi }{\left[\begin{array}{ccc}{{{{\rm{e}}}}}^{-\frac{1}{c}[D{f}_{1}^{2}+{c}^{2}{\mu }_{a}]{t}_{1}} & \cdots & {{{{\rm{e}}}}}^{-\frac{1}{c}[D{f}_{M}^{2}+{c}^{2}{\mu }_{a}]{t}_{1}}\\ \vdots & \ddots & \vdots \\ {{{{\rm{e}}}}}^{-\frac{1}{c}[D{f}_{1}^{2}+{c}^{2}{\mu }_{a}]{t}_{M}} & \cdots & {{{{\rm{e}}}}}^{-\frac{1}{c}[D{f}_{M}^{2}+{c}^{2}{\mu }_{a}]{t}_{M}}\end{array}\right]}_{M\times M}{\left[\begin{array}{c}\overline{\varPhi }({f}_{1})\\ \vdots \\ \overline{\varPhi }({f}_{M})\end{array}\right]}_{M\times 1},$$

where *M* is the number of time bins, and $A$ is a sparse matrix representing the transformation process. Here, Quasi-minimal residual method, Preconditioned Conjugate Gradient (PCG), or Generalized Minimal Residual (GMRES) algorithms can be used to solve this inverse problem. Then $\phi \left(x,y,z=0,t\right)$ can be obtained by using IFFT over ${k}_{x}$ and ${k}_{y}$ and numerical transform over $f$.

## Supplementary information

Supplementary Information

## Data Availability

The data that support the findings of this study are available from the corresponding authors upon request.

## Code availability

The code for the implementation used in this paper is available from the corresponding authors upon request.
