# Supplementary material for: A boundary migration model for imaging within volumetric scattering media
Source: Nat Commun. 2022 Jun 9;13:3234. doi: 10.1038/s41467-022-30948-7 (PMC9184484; doi:10.1038/s41467-022-30948-7)
Supplement: Supplementary file 1 — Supplementary Information [file 41467_2022_30948_MOESM1_ESM.pdf]

Supplementary Information for

A boundary migration model for imaging within volumetric scattering media

Dongyu Du, Xin Jin, Rujia Deng, Jinshi Kang, Hongkun Cao, Yihui Fan, Zhiheng Li, Haoqian Wang, Xiangyang Ji & Jingyan Song

This PDF file includes:

Supplementary Note 1:
Equipment details and imaging scenarios
Supplementary Note 2:
Estimation of the scattering characteristics of scattering media
Supplementary Note 3:
Validity analysis of the algorithm
Supplementary Note 4:
Evaluation of the resolution in the polyethylene foam scattering scenario
Supplementary Note 5:
Reconstructions of objects with different albedo surfaces
Supplementary Note 6:
Reconstructions of objects under ambient illumination
Supplementary Note 7:
Comparison of relevant reconstruction methods
Supplementary Note 8:
Discussion of depth reconstruction
Supplementary Figures 1 to 14
Supplementary Tables 1 to 9

Supplementary Note 1: Equipment details and imaging scenarios

As shown in Supplementary Fig. 1a, our hardware imaging system consists of a 20fs pulsed laser (Coherent Vitara-T), a 32 × 32 array of single-photon avalanche diode detector (Photon Force PF32, $6.95\mu m$ ⌀ active area, $50\mu m$ pixel pitch), several controlling electronics and optical elements used for scanning and focusing. Supplementary Fig. 1b shows the optical path of the confocal configuration used in our system. The laser hand emits a collimated light with a wavelength of 808nm at an 80MHz repetition rate and 580mW average power. Then the light passes through a polarized beam splitter cube (Thorlabs PBS255) and is incident into the scene after being reflected by a 2-axis scanning galvanometer (Thorlabs GVS012) controlled with a data acquisition device (NI-DAQ USB-6343). Light returning from the scenario along the same optical path is focused to the detector by a lens with 8mm focal length and 1.4 maximal f-number. The delayer is used to reshape the synchronization signal output from the laser into a standard Transistor-Transistor Logic (TTL) signal, which is then used as the acquisition trigger signal of the single-photon avalanche diode (SPAD). Since the trigger signal frequency of the detector is 80MHz and its temporal resolution is 55ps, the temporal dimension of the data collected by the SPAD is 230. Therefore, the SPAD array captures the data with a size of 32 × 32 × 230 for each scanning point. We select several pixels in the array that are less affected by the initial bright light caused by the internal reflection of the confocal optical path. Then these pixels are averaged to generate the measurement of the current scanning point. By controlling the angle of the galvanometer, the system scans a grid of 64 × 64 points on the front surface of the media so that the detector collects 3D data: two spatial dimensions with the size of the scanning grid and one temporal dimension of 230 bins.

We evaluate the reconstruction performance of our algorithm in two typical scattering scenarios: 2D and 3D Lambertian objects embedded in the polyethylene foam and the fog. The two scattering imaging scenarios are shown in Supplementary Fig. 2-3. The characteristics, including scattering, absorption, and propagation angle of two scattering media, are quite different and are estimated in Supplementary Note 2. Polyethylene foam is a homogeneous static isotropic scattering medium while the fog is a dynamic forward scattering medium[1-3]. They produce different temporal responses as shown in Fig. 3b and 4b in the main text. The former is affected by the backscattered light so

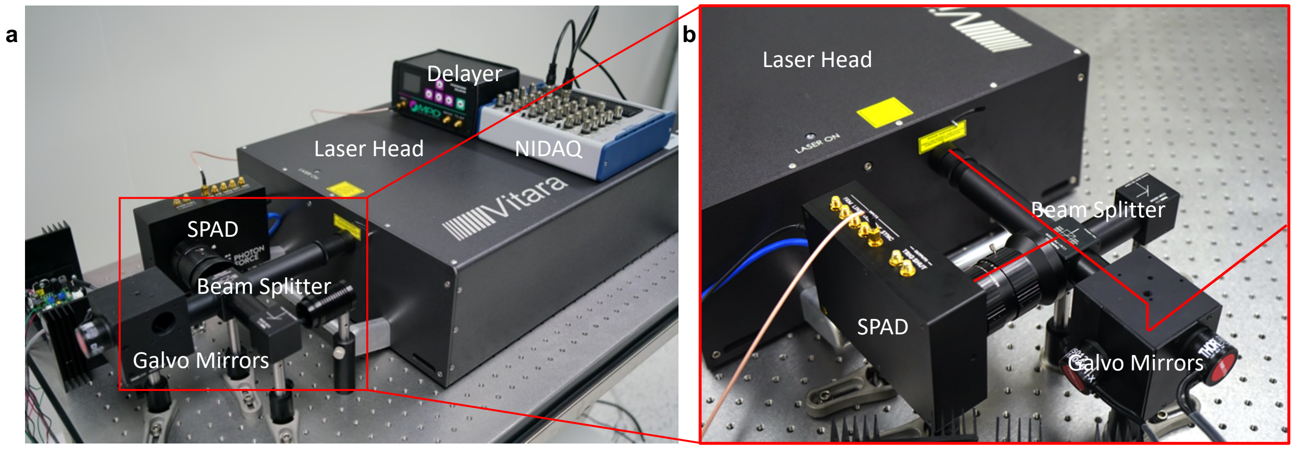

Supplementary Fig. 1 Hardware prototype and optical path illustration. a The main components of the setup are a pulsed laser, a SPAD array, a delayer used for sync signal shaping, a data acquisition device used for controlling, and some optical elements including beam splitter, galvo mirrors, and lens used for scanning and focusing. **b** The confocal optical path illustration: the pulsed laser and the SPAD share the optical path through the beam splitter. By controlling the angle of the galvo mirrors, the system scans different points on the front surface of the scattering media.

that signal photons are highly coupled with background photons. The latter is affected by the time variability of the fog and thus the temporal distribution of signal photons is superimposed with dynamic noise. Both cases exacerbate the reconstruction difficulty while we demonstrate the ability of our method in these two scattering scenarios.

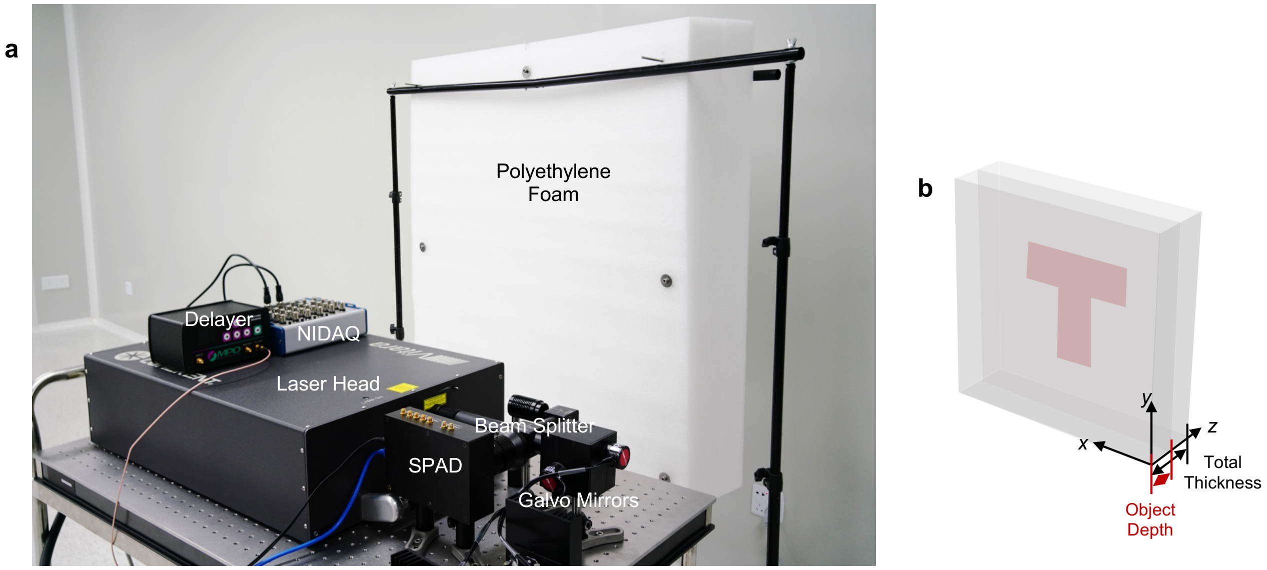

Supplementary Fig. 2 Photograph and layout of the polyethylene foam scattering scenario. a Confocal imaging system captures the photons reflected from the foam scenario. **b** The total thicknesses of the foam range from 12cm to 25cm. The object is embedded in the foam and its depth is defined as the distance from the front surface of the scattering media to the object.

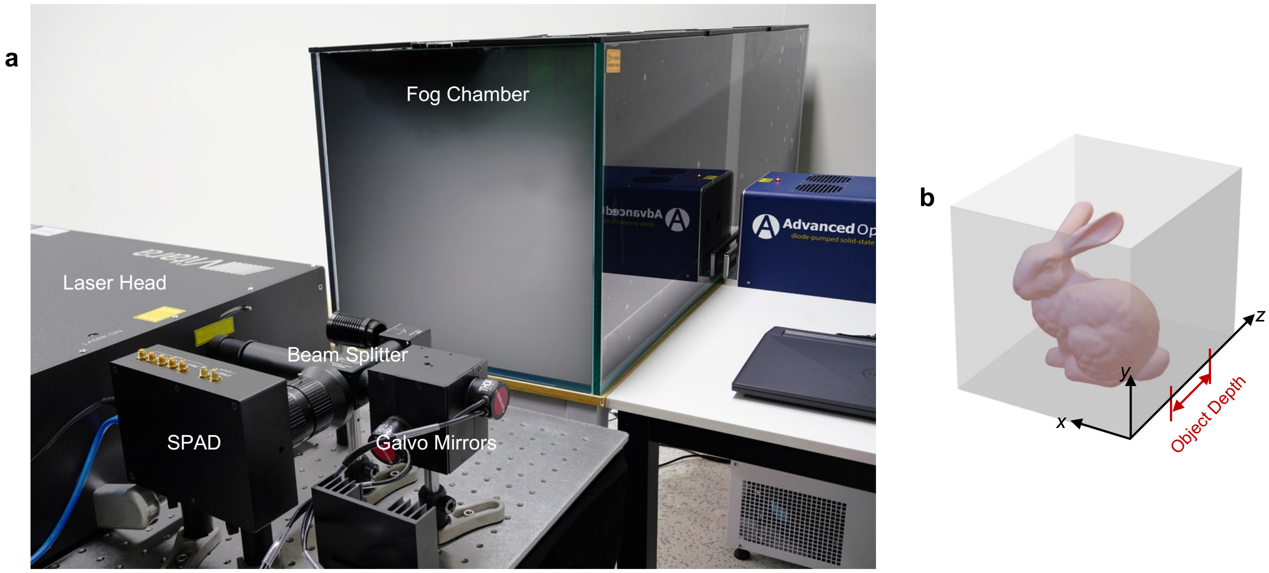

Supplementary Fig. 3 Photograph and layout of the fog scattering scenario. a An artificial fog chamber with a size of 50cm × 50cm × 150cm provides the dynamic scattering environment. **b** The object is embedded in the fog and its depth range is defined as the distance range from the front surface of the scattering media to the object.

Supplementary Note 2: Estimation of the scattering characteristics of scattering media

In this section, the scattering characteristics of the polyethylene foam and the fog are estimated using the solution of the diffusion equation for the slab media and Beer-Lambert Law, respectively. The former provides the optimal reduced scattering coefficient μ_s' and absorption coefficient μ_a of the polyethylene foam. The reduced scattering coefficient is given by $\mu_s' = \mu_s(1-g)$, where μ_s is the scattering coefficient and $g \in [-1,1]$ is a parameter of Henyey-Greenstein phase function that describes the propagation angle of the scattering particles. Positive g indicates the forward scattering and negative g indicates the backward scattering. Polyethylene foam is a homogeneous static isotropic scattering medium with $g = 0$, where the photon scatters in all angles with equal probability, while the fog is a dynamic forward scattering medium[1-3] with $g \approx 0.85$. The Beer-Lambert Law gives the estimation of the transport coefficient μ_t of the fog, which is defined as $\mu_t = \mu_s + \mu_a$.

Estimating the scattering characteristic of polyethylene foam. We estimate the reduced scattering coefficient μ_s' and absorption coefficient μ_a of the polyethylene foam by optimizing the residual error between the measured temporal response and the theoretical solution of the diffusion equation[4, 5] (Eq. (1) in the main text) for the slab media which is given by:

$$\phi(t,\rho) = \frac{\exp(-\mu_a ct - \frac{\rho^2}{4Dct})}{2(4\pi Dc)^{3/2}t^{5/2}} \sum_{m=-\infty}^{+\infty} \left[(d - z_{+,m})\exp\left(-\frac{(d-z_{+,m})^2}{4Dct}\right) - (d - z_{-,m})\exp\left(-\frac{(d-z_{-,m})^2}{4Dct}\right) \right], \quad (1)$$

where $\phi(t,\rho)$ represents the power that photons enter at the origin of the coordinate system at $t = 0$, and exit from the far boundary of the scattering media at time t per unit of area and unit of time. ρ is the distance from the exit

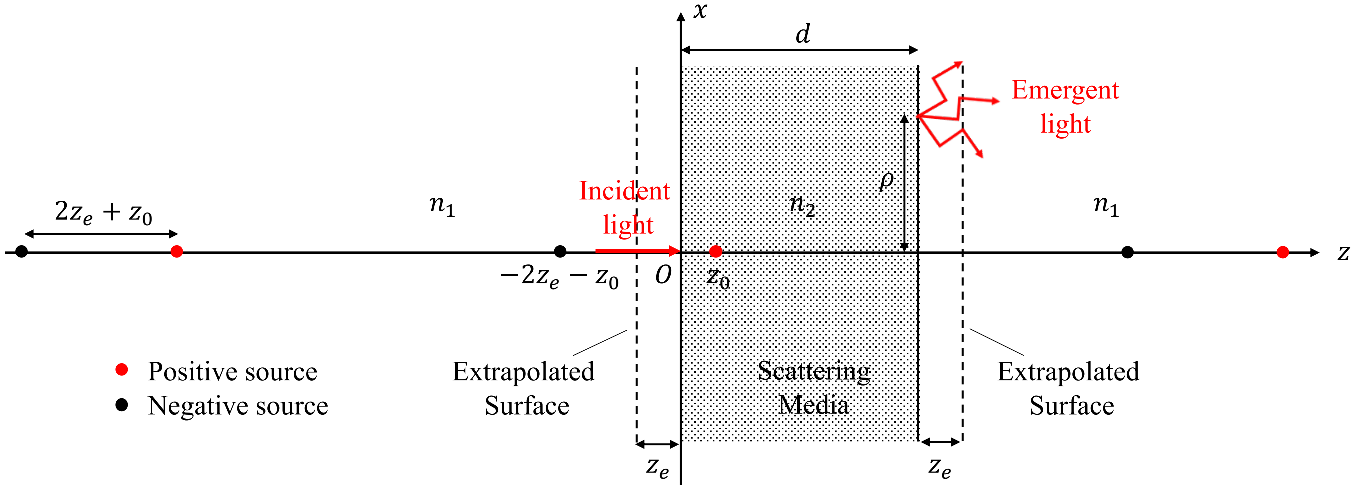

Supplementary Fig. 4 Scattering media with slab geometry and some notations. The dashed lines are the extrapolated surfaces of the media where the diffusive intensity is zero. z_0 represents the location where incident light is initially scattered isotropically. Red points and black points represent the positions of positive and negative sources, respectively. ρ is the distance from the exit point to the plane $x = 0$. d is the thickness of the scattering media. n_1 and n_2 are the refractive indices of light in external media and scattering media, respectively.

point to the plane $x = 0$ as shown in Supplementary Fig. 4. Additionally, diffusion coefficient D is given by $D = (3(\mu_s' + \mu_a))^{-1}$, c is the speed of the light and d is the thickness of the scattering media. Supplementary Equation (1) is obtained with two assumptions: (1) incident light is initially scattered isotropically at a distance of $z_0 = 1/\mu_s'$ in the scattering media[6]; (2) the average diffuse intensity is assumed to be zero at two extrapolated flat surfaces located at a distance, z_e, away from either boundary of the scattering media. To satisfy these boundary conditions, an infinite number of positive and negative dipole sources can be virtually placed in the scenario at the following positions[5]:

$$\begin{aligned} z_{+,m} &= 2m(d + 2z_e) + z_0, \\ z_{-,m} &= 2m(d + 2z_e) - 2z_e - z_0, \\ m &= 0, \pm 1, \pm 2, \ldots \end{aligned} \tag{2}$$

In general, 7 dipoles are sufficient to maintain the truncation error at a negligible level[5]. The extrapolated distance z_e is given by:

$$z_e = \frac{2}{3\mu_s'} \frac{1 + 3\int_0^{\frac{\pi}{2}} R(\theta_i)\cos^2(\theta_i)\sin(\theta_i)d\theta_i}{1 - 2\int_0^{\frac{\pi}{2}} R(\theta_i)\cos(\theta_i)\sin(\theta_i)d\theta_i}, \tag{3}$$

where θ_i is the angle of incidence light, $R(\theta_i)$ is the reflection coefficient calculated by the Fresnel reflection coefficients[7]. For a given θ_i, the reflection coefficient $R(\theta_i)$ for unpolarized light is given by:

$$R(\theta_i) = \frac{1}{2}\left\{\left[\frac{n\cos(\theta_i) - \cos(\theta_t)}{n\cos(\theta_i) + \cos(\theta_t)}\right]^2 + \left[\frac{\cos(\theta_i) - n\cos(\theta_t)}{\cos(\theta_i) + n\cos(\theta_t)}\right]^2\right\}, \tag{4}$$

where $n = n_2/n_1$ is the relative refractive index and is defined as the ratio between the refractive index of the scattering media and that of the external media, as shown in Supplementary Fig. 4. $\theta_t = \arcsin\left[n \cdot \sin\left(\theta_i\right)\right]$ is the refracted angle.

Therefore, there are three parameters of scattering media to be estimated: reduced scattering coefficient μ_s', absorption coefficient μ_a and relative refractive index n. The pulsed light is used to illuminate one side of the polyethylene foam, and the SPAD array is used to capture the temporal response $\varphi(t)$ at the other side. For simplicity, the detection point is set at $x = 0$ then the distance ρ is equal to 0. We measure 9 different thicknesses, d, of the polyethylene foam, ranging from 2cm to 10cm with an increment of 1cm. Given a fixed value of the relative refractive index n_0, we optimize the μ_s' and μ_a to minimize the following objective function:

$$\underset{\mu_s', \mu_a}{\arg\min} \sum_{i=1}^{k} \frac{1}{k}\left[\varphi(t, d_i) - \phi(t, \rho, d_i, n_0)\right]^2. \tag{5}$$

The Levenberg-Marquardt algorithm is used to iteratively solve this non-linear optimization in MATLAB. There are a couple of factors that need to be taken into account before the optimization. The first one is the scale of the measured temporal response which depends on the power of the incident light. So, the measurements and the theoretical values are normalized to a maximum of 1. The second one is the experimental delay between triggering the laser and the photons being captured by the detector, which depends on the optical path between the laser and the detector and the time jitter of the system. So, we need to shift the measurements to produce a good fit of the model. First, we calibrate the distance between the laser and detector. Then the offset value of each measurement is also regarded as an optimization parameter. In this case, the number of the optimization parameter in our experiment is 11, including the reduced scattering coefficient, absorption coefficient, and 9 offsets of the measurements with different thicknesses of the polyethylene foam.

Empirically given the relative refractive index across a range from 1.12 to 1.32 with an increment of 0.01, the initializations of μ_s' from 2.0cm^{-1} to 3.0cm^{-1} with an increment of 0.1cm^{-1} and μ_a equals 0, a scatterplot of the mean squared errors (MSE) between the model and all measurements is shown in Supplementary Fig. 5a. It can be found that the relative refractive index of 1.23 produces the minimal MSE with the initializations of $\mu_s' = 2.6\text{cm}^{-1}$ and $\mu_a = 0\text{cm}^{-1}$, in which case the optimal parameters are given by $\mu_s' = 3.1377\text{cm}^{-1}$ and $\mu_a =$

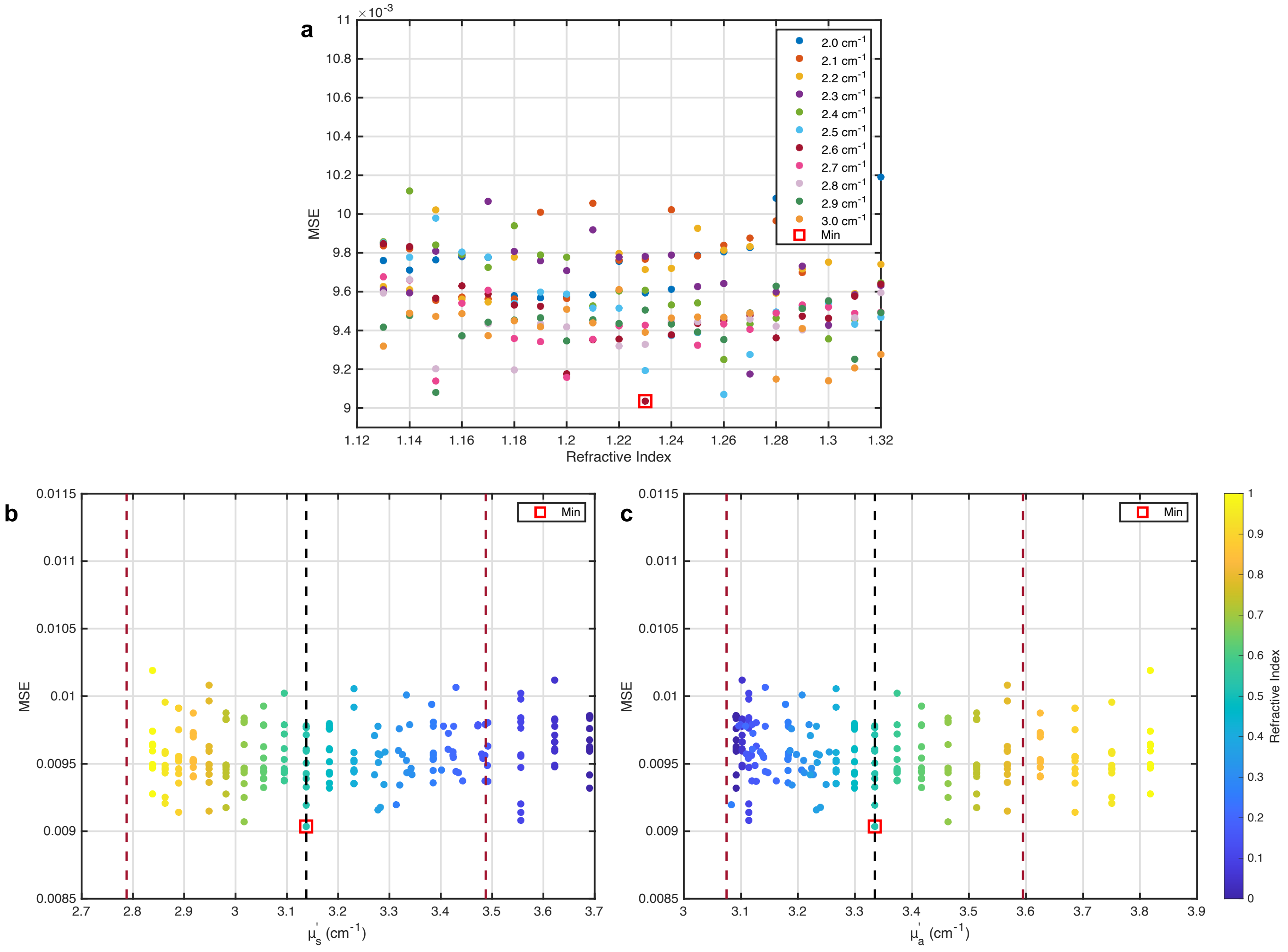

Supplementary Fig. 5 Optimization results of the parameters. The scattering characteristics of the polyethylene foam are estimated by comparing the measured temporal response with the theoretical model given by the solution of the diffusion equation for slab geometry, Supplementary Equation (1). We run optimizations with the relative refractive index ranging from 1.12 to 1.32 with an increment of 0.01. For a given relative refractive index, we optimize the value with different initializations of the reduced scattering coefficient, from 2.0cm^{-1} to 3.0cm^{-1} with an increment of 0.1cm^{-1}, so that we run a total of 220 optimizations. **a** The mean square error of the optimizations indicates that a relative refractive index of 1.23 produces the minimal MSE with the initialization of the reduced scattering coefficient equal to 2.6cm^{-1}. The colors give the initializations of the reduced scattering coefficient. **b-c** The mean square error of all optimizations against the reduced scattering coefficient and the absorption coefficient, respectively. The black dashed lines show the optimal parameters and the red dashed lines indicate the boundary range which contains 80% of the optimized values. The colors give the values of the relative refractive index.

$3.3348 \times 10^{-2}\text{cm}^{-1}$. As shown in Supplementary Fig. 6, these parameters produce a good fit to the measured temporal responses in all thicknesses of the polyethylene foam. To quantify the uncertainty of the estimated parameters, we analyze the MSE against the reduced scattering coefficient and the absorption coefficient for all optimizations, as shown in Supplementary Fig. 5b-c. The black dashed lines indicate the optimal parameters and the red dashed lines show the boundary of the range containing 80% of all estimated values. Therefore, the reduced scattering coefficient μ_s' and the absorption coefficient μ_a of the polyethylene foam used in our experiments are estimated as: $\mu_s' = 3.1377 \pm 0.35\text{cm}^{-1}$ and $\mu_a = 3.3348 \times 10^{-2} \pm 2.6 \times 10^{-3}\text{cm}^{-1}$.

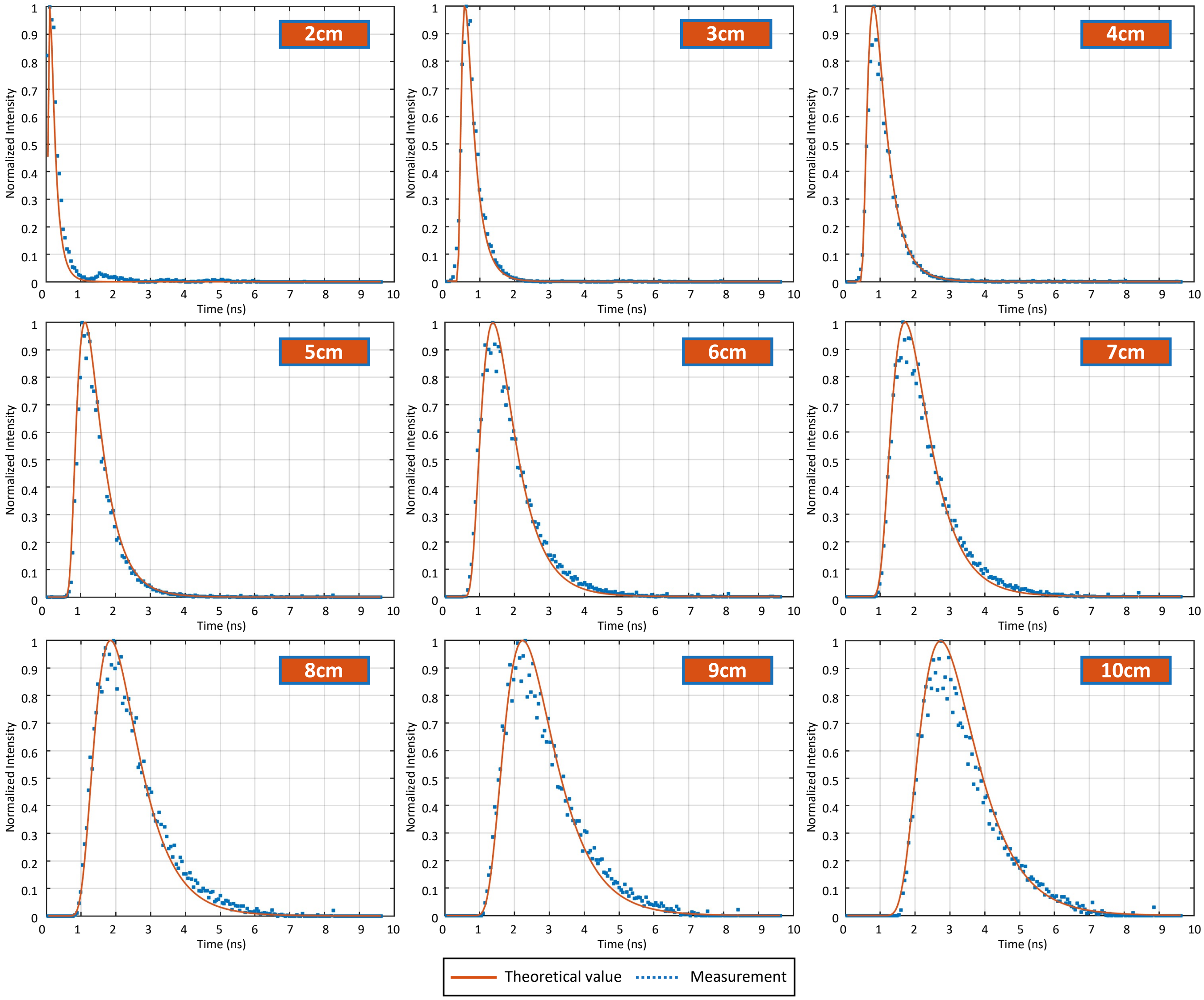

Supplementary Fig. 6 The fitting results of the measured temporal responses and the theoretical values. We capture 9 measurements of the polyethylene foam with different thicknesses, ranging from 2cm to 10cm with an increment of 1cm. The orange lines are the theoretical values and the blue dashed lines are the measurements. In all cases, the parameters, $\mu_s' = 3.1377\text{cm}^{-1}$ and $\mu_a = 3.3348 \times 10^{-2}\text{cm}^{-1}$, produce a good fit between the measurements and the theoretical model.

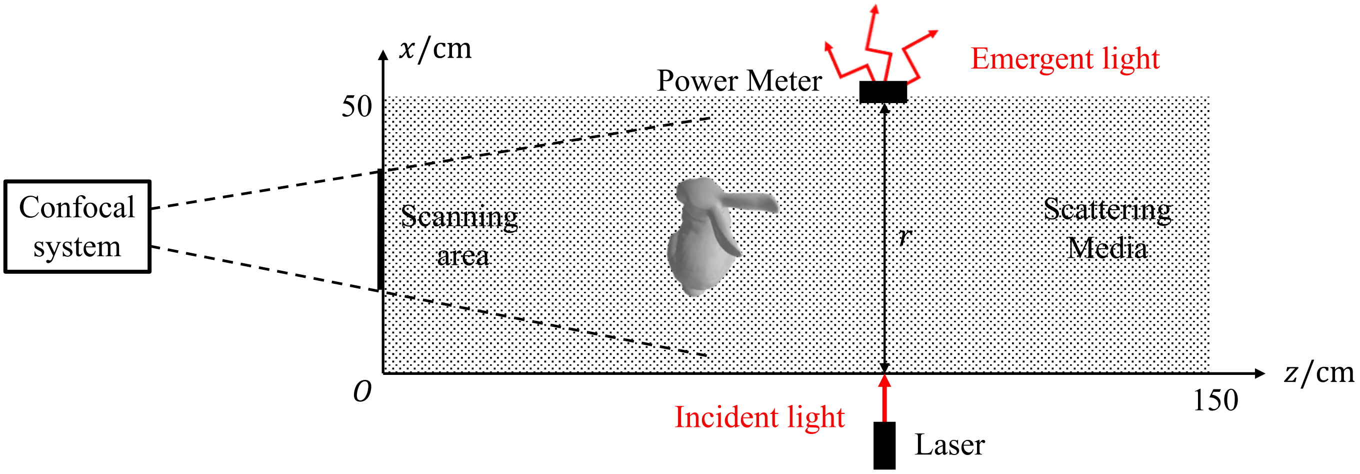

Supplementary Fig. 7 Schematic of the setup used for the fog transmittance measurements. A laser emits the light into the scattering media on one side of the fog chamber. A power meter detects the optical power of the emergent light on the other side of the fog chamber. The distance r between the laser and power meter is 50cm. The size of the fog chamber is 50cm × 50cm × 150cm. The scanning area of the confocal system is 25cm × 25cm. Traveling along the direction of the light, the scanning range of the depth plane where the object located is roughly 45cm × 45cm.

Estimating the scattering characteristic of the fog. The light propagates in the fog is attenuated by the scattering and absorption of the scattering particles. Both effects can be considered as a single transport coefficient μ_t, which is defined as the sum of the scattering coefficient μ_s and the absorption coefficient μ_a. According to the Beer-Lambert Law, if the light propagates a distance r with the transport coefficient μ_t, the optical power is given by:

$$P(r) = P_0 e^{-\mu_t r}, \tag{6}$$

where P_0 is the initial power of the incident light and $\mu_t r$ gives the multiples of the mean free paths (MFP, defined as $1/\mu_t$), also known as optical thickness T. In this case, the transport coefficient can be estimated by optical power measurements. The transmittance measurements are retrieved as shown in Supplementary Fig. 7, the laser emits the light with different initial optical powers into the scattering media on the one side of the fog chamber while a power meter detects the outgoing power of the light through the fog, r = 50cm, on the other side of the scattering media. We measure 10 outgoing optical powers at different initial optical powers, as shown in Supplementary Table 1. The calculated 10 transport coefficients are averaged to obtain the final estimated value: $\mu_t = 0.2403\text{cm}^{-1}$.

P_0 (mW)	676	697	723	752	776	802	822	849	878	903
P_r (mW)	0.0054	0.0061	0.0055	0.0038	0.0064	0.0039	0.0038	0.0047	0.0044	0.0042
$\mu_t = \frac{1}{r}\log\frac{P_0}{P_r}$ (cm^{-1})	0.2347	0.2328	0.2356	0.2438	0.2340	0.2449	0.2456	0.2422	0.2441	0.2457
Avg. μ_t (cm^{-1})	0.2403									

Supplementary Table 1. Estimation of the transport coefficient of the fog. Given 10 different initial optical powers, we measure outgoing optical powers using the setup in Supplementary Fig. 7. The 10 transport coefficients are averaged as 0.2403cm^{-1}, which is the final estimated transport coefficient of the fog.

Supplementary Note 3: Validity analysis of the algorithm

In this section, we analyze the validity range of our algorithm. The proposed BMM algorithm is based on the diffusion equation. It treats light propagating through the scattering media as a random walk[4] so that photons diffused inside the scattering media follow some defined probability rules, which can be regarded as the quantitative indications to determine whether the diffusion approximation breaks down. In particular, the average arrival time of photons is one of the quantitative indications. The average arrival time, described as the mean time required by a photon going through the scattering media, is computed by[8]

$$\Delta t = \frac{\int_0^\infty t' I(t') dt'}{\int_0^\infty I(t') dt'}, \tag{7}$$

where $I(t')$ is the temporal response measured experimentally. The theoretical prediction of the average arrival time for a photon travels a distance d is proportional to $d^2/6Dc$, where D is the diffusion coefficient, and c is the speed of light[8-10]. Considering the influence of the experimental factors such as the time duration of the incident pulse, the linear relationship between measured temporal displacement and theoretical average arrival time is given by[11]:

$$\Delta t = \alpha \frac{d^2}{6Dc}, \tag{8}$$

where α is a proportionality factor that depends on the short pulse duration and so on. We analyze the linear relationship between the measured average arrival time and the theoretical value to verify that all our experiments are

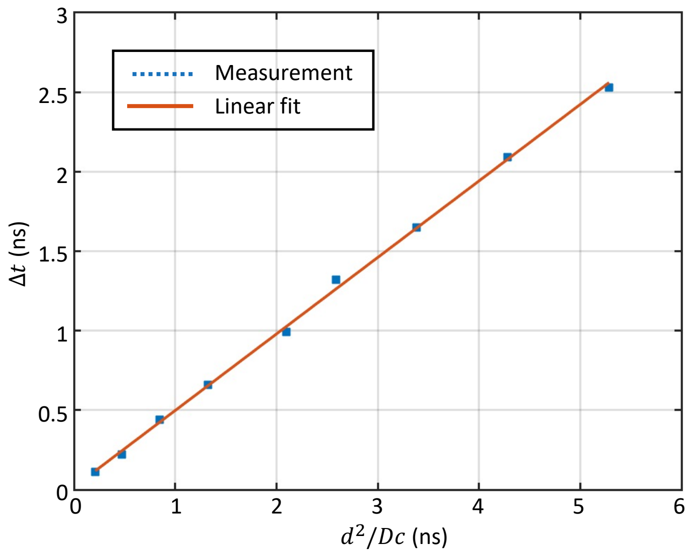

Thickness (cm)	2	3	4	5	6	7	8	9	10
Δt (ns)	0.1100	0.2200	0.4400	0.6600	0.9900	1.3200	1.6500	2.0900	2.5300
$\frac{d^2}{6Dc}$ (ns)	0.2114	0.4757	0.8456	1.3213	1.9027	2.5897	3.3825	4.2810	5.2852

Supplementary Fig. 8 The linear relationship between the measured average arrival time and the theoretical value. The relationship between the peak shift of the measured temporal response Δt and theoretical average arrival time $d^2/6Dc$ is fitted as a linear function. The proportionality factor α is linearly fitted as 0.4812.

within the validity range of the diffusion equation. We measure the temporal responses in the polyethylene foam with 9 different thicknesses ranging from 2cm to 10cm with an increment of 1cm. The measured temporal responses are shown in Supplementary Fig. 6. Then the peak shifting time of each temporal response is approximated as the measured average arrival time Δt here[11]. The fitted result indicates that measured Δt is a linear function of the theoretical value $d^2/6Dc$, depicted by the orange curve in Supplementary Fig. 8, where the proportionality factor α is 0.4812. It demonstrates that all the experiments are within the validity range of the diffusion equation.

Several studies show that the diffusion equation models the scattered light more accurate with the increase of the imaging depth[8, 12, 13]. Therefore, the validity range of our algorithm is theoretically unbounded. However, in practical experiments, the imaging quality is limited by the signal-to-background ratio (SBR) of measurement which depends on the characteristics of the physical setup, including the incident power of the laser, the sensitivity of the detector, the albedo of the object surface and so on. To quantitatively analyze the proportion of signal photons and the scattering strength in our experiments, we calculate the SBR of measurements. The SBR is defined as the ratio of signal photon intensity to background photon intensity and given by:

$$SBR = \frac{\| I - I_b \|_2^2}{\| I_b \|_2^2}, \tag{9}$$

where I is the intensity of photons reflected by both the object and the scattering media, and I_b is the intensity of background photons reflected by the scattering media. Then, the intensity of signal photons can be calculated by $I - I_b$. We capture 9 measurements with objects embedded in the polyethylene foam at different depths, ranging from 2cm to 10cm with an increment of 1cm, to obtain the intensity of all the photons. Corresponding to the different object depths, the total thickness of the foam varies from 9cm to 17cm. The measured temporal responses are shown as the orange lines in Supplementary Fig. 10. Then we take the object out and measure the temporal responses to obtain the

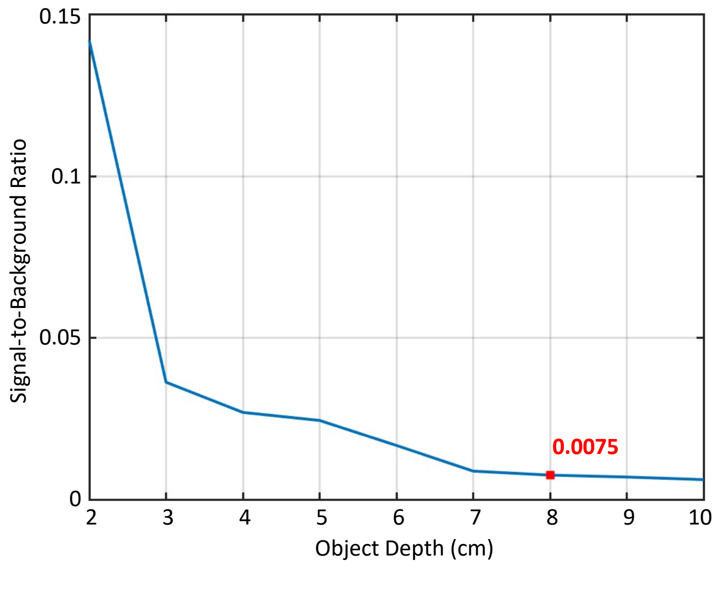

Supplementary Fig. 9 The relationship between the SBR and the object depth in the polyethylene foam. The SBR of the measurements at different depths can be obtained using Supplementary Equation (9). The results indicate that the signal photons decrease with the increase of the object depth. Using our setup, the maximum imaging depth of our algorithm to reconstruct the Lambertian object is 8cm where the SBR of the measurement is only 0.75%.

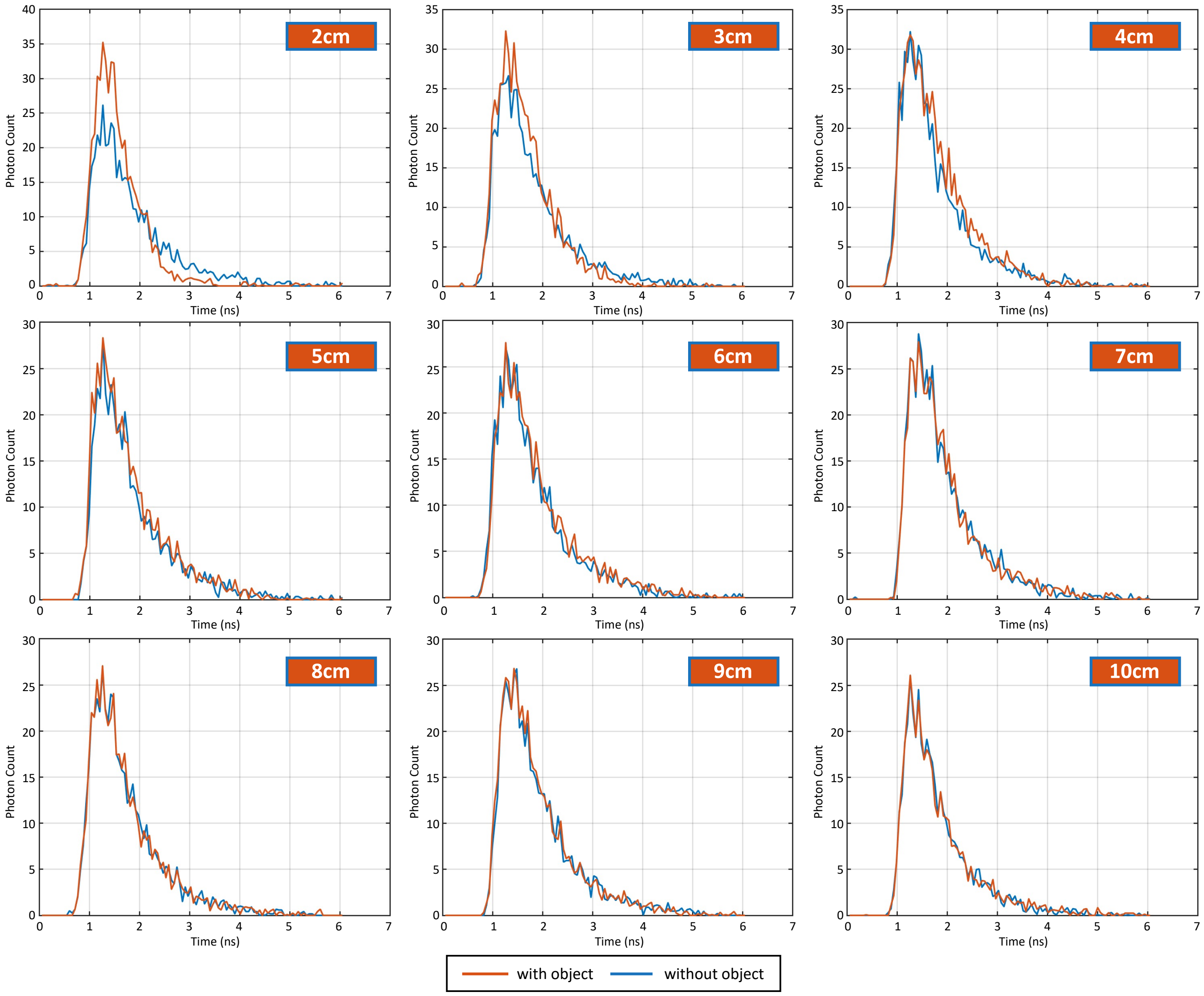

Supplementary Fig. 10 Comparison of the temporal response captured in the polyethylene foam with and without objects. The measurements are captured with different object depths in the polyethylene foam, ranging from 2cm to 10cm with an increment of 1cm. The orange lines show the photons reflected by both object and scattering media, while the blue lines show the photons reflected only by the scattering media. The difference between them indicates the intensity of the signal photons reflected by the object.

---

background intensity, the blue lines in Supplementary Fig. 10. It can be seen that the difference between the two curves decreases with the increase in the object depth, which indicates the decrease of the signal photons. Using Supplementary Equation (9), we calculate the SBR of each measurement and find that the SBR is a decreasing function of the object depth, as shown in Supplementary Fig. 9.

The experiments are conducted with Lambertian objects at different transport mean free paths (TMFPs) to evaluate the validity range of our algorithm (The scattering lengths described below are all calculated in one direction). As a comparison, the reconstructions of the time gating algorithm are also presented in Supplementary Table 2. Since the time gating depends on isolating the signal photons, it can only work at less than 9.5 TMFPs and fails to handle the cases where the signal photons are too weak to be separated, such as the cases at a depth of 4cm to 10cm shown

in Supplementary Fig. 10. In contrast, benefitting from the accurate modeling and efficient use of all the optical photons, our algorithm performs well even when the object is at a depth of 25.4 TMFPs where the SBR is only 0.75%, as marked by the red dot in Supplementary Fig. 9.

More importantly, it is found that the upper bound of the validity range of our algorithm can be improved further when we improve the SBR by increasing the incident power of the laser, using more sensitive photon detectors or enhancing the albedo of the object surface. For example, we use retroreflective objects to increase the signal intensity and test the algorithm's limit again under this condition, as shown in Supplementary Table 3. Due to the increase of

Lambertian Object	Depth	2cm<br>6.3 TMFPs	3cm<br>9.5 TMFPs	4cm<br>12.7 TMFPs	5cm<br>15.9 TMFPs
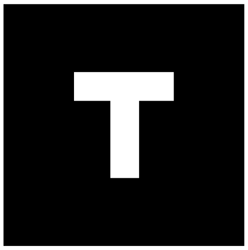	Time gating	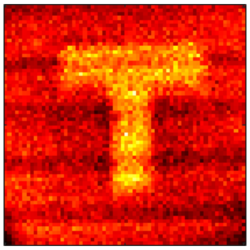	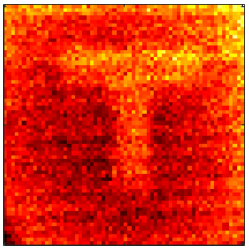	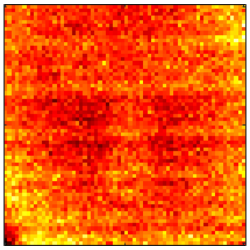	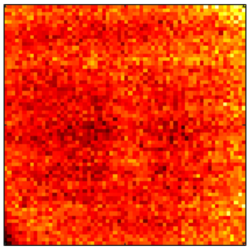
	Ours	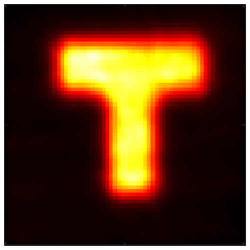	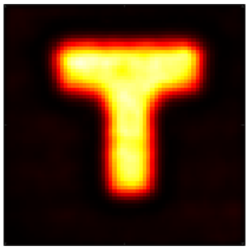	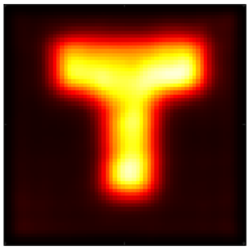	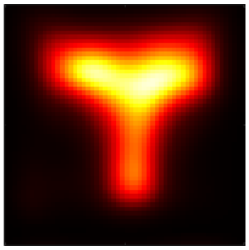
	Depth	6cm<br>19.0 TMFPs	7cm<br>22.2 TMFPs	8cm<br>25.4 TMFPs	9cm<br>28.5 TMFPs
	Time gating	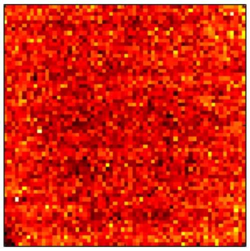	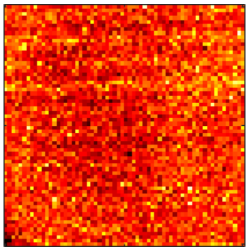	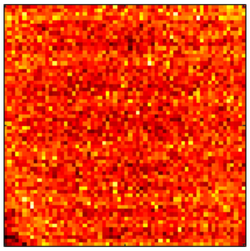	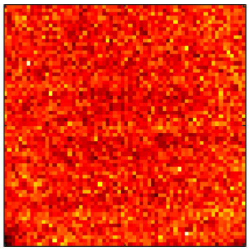
	Ours	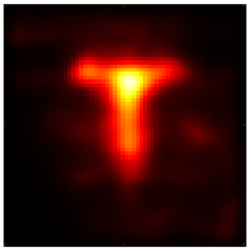	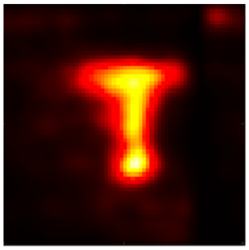	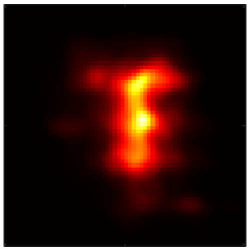	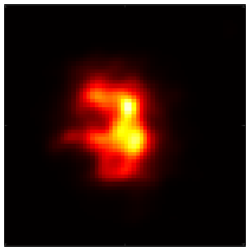

Supplementary Table 2. Comparison of time gating and our algorithm for reconstructing Lambertian objects. We compare the results of time gating and our algorithm for reconstructing a Lambertian letter 'T' at different depths, ranging from 2cm to 9cm with an increment of 1cm. It can be found that time gating can only reconstruct the object at a depth less than 9.5 TMFPs, while our algorithm works even at 25.4 TMFPs.

signal intensity, time gating can reconstruct the object at a depth less than 12.7 TMFPs, while our algorithm successfully reconstructs the object at a depth of 28.5 TMFPs.

To summarize, we demonstrate that all our experiments are within the validity range of the diffusion equation. Additionally, the validity range of our algorithm is theoretically unbounded but related to the SBR of the measurements in practical experiments. Using our present experimental setup, the maximum depth of the proposed algorithm to reconstruct the Lambertian and retroreflective object is 25.4 TMFPs and 28.5 TMFPs, respectively, i.e., the round-trip optical paths are 50.8 TMFPs and 57.0 TMFPs, respectively.

Retroreflective Object	Depth	2cm 6.3 TMFPs	3cm 9.5 TMFPs	4cm 12.7 TMFPs	5cm 15.9 TMFPs
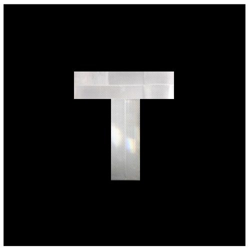	Time gating	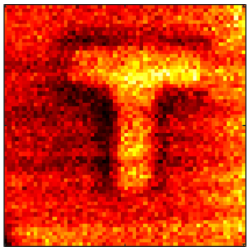	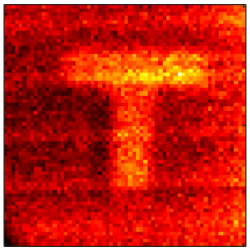	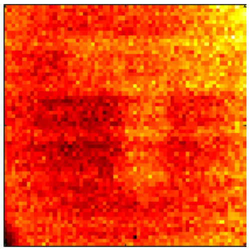	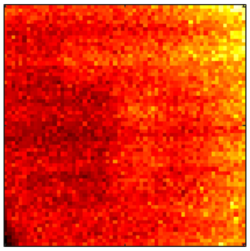
	Ours	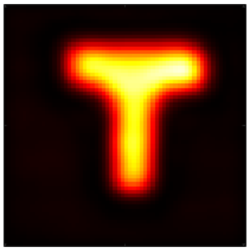	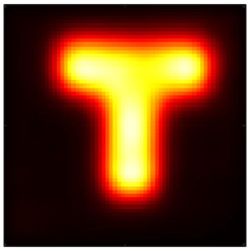	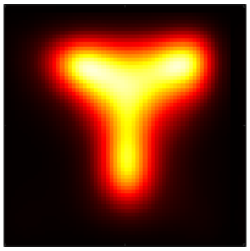	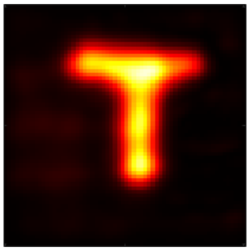

Depth	6cm 19.0 TMFPs	7cm 22.2 TMFPs	8cm 25.4 TMFPs	9cm 28.5 TMFPs	10cm 31.7 TMFPs
Time gating	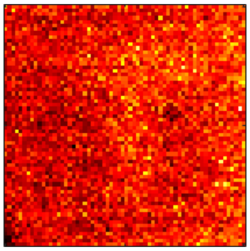	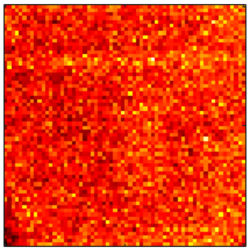	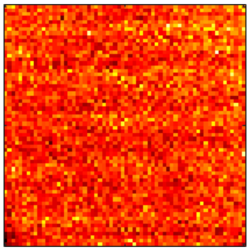	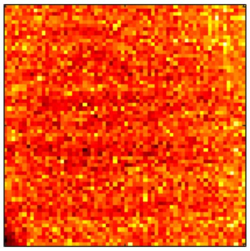	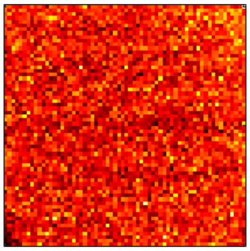
Ours	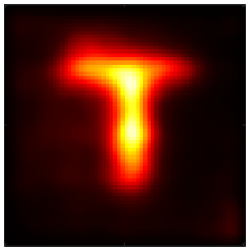	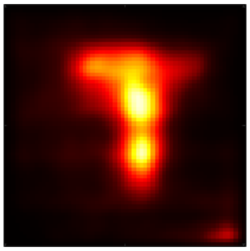	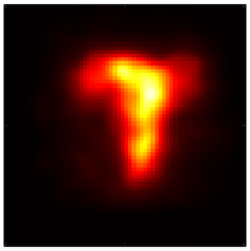	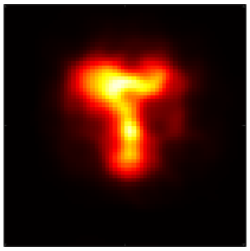	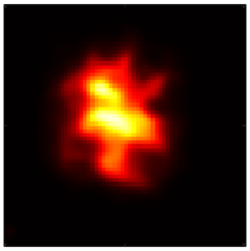

Supplementary Table 3. Comparison of time gating and our algorithm for reconstructing retroreflective objects. We compare the results of time gating and our algorithm for reconstructing a retroreflective object at different depths, ranging from 2cm to 10cm with an increment of 1cm. It can be found that time gating can only reconstruct the object at a depth less than 12.7 TMFPs, while our algorithm works even at 28.5 TMFPs.

Supplementary Note 4: Evaluation of the resolution in the polyethylene foam scattering scenario

In this section, we derive and evaluate the resolution of our system in the polyethylene foam scattering scenario. The resolution is the minimal distinguishable distance Δx between two points x_1 and x_2, as shown in Supplementary Fig. 11a. When the scanning interval is small enough that all diffused spots can cover the entire scanning area, the key factor affecting the resolution is the diffusion effect caused by the scattering media. In our analysis, we neglect the difference in axial propagation between the two points, and thus the scattering media primarily affect the lateral diffusion of the light. The lateral resolution of the system is derived using the full width at half maximum (FWHM) criterion.

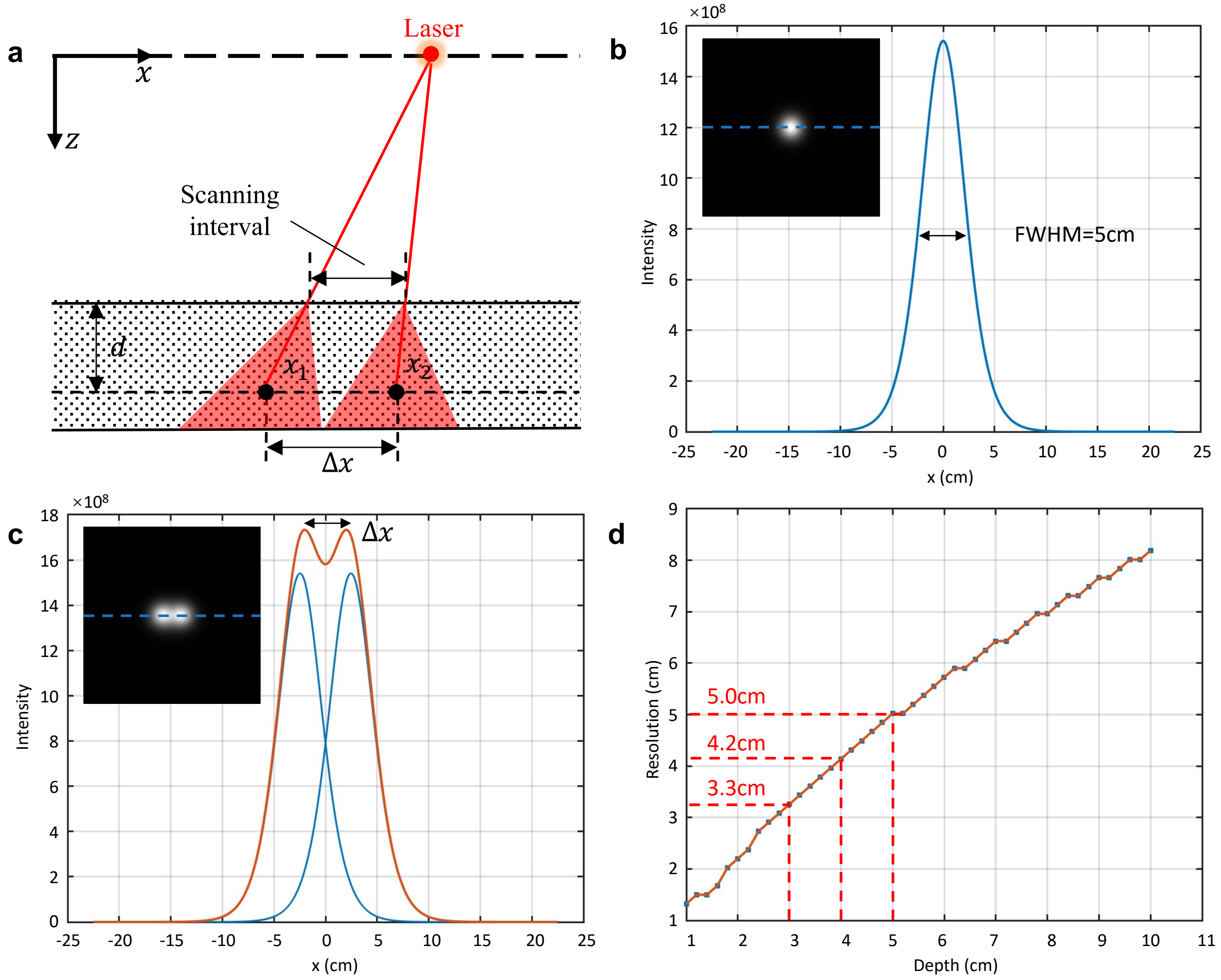

Supplementary Fig. 11. Illustration of the resolution analysis. a For a given depth d, the minimal distinguishable distance Δx between two points x_1 and x_2 is determined by the diffusion effect. **b** A full width at half maximum of a single diffusion spot measured at a depth of 5cm. The subfigure is the spatial distribution of a single diffusion spot. **c** Two points can be resolved if their distance is larger than Δx. The subfigure is the spatial distribution of double diffusion spots. **d** Resolution is calculated by Supplementary Equation (11) and is a function of depth. The theoretical resolutions at the depths of 3cm, 4cm, 5cm are 3.3cm, 4.2cm and 5.0cm, respectively.

For a given depth d, the propagation of the scattered light $\phi(x, y, t)$ can be calculated by the theoretical solution of diffusion equation as shown in Supplementary Equation (1). Then the spatial distribution of the laterally diffused spot $\bar{\phi}(x, y)$ is given by:

$$\bar{\phi}(x, y) = \sum_{t=1}^{n} \frac{1}{n} \phi(x, y, t), \tag{10}$$

where n is the length of the time bins. The subfigure in Supplementary Fig. 11b shows the spatial distribution of diffused spots at a depth of 5cm, and the blue curve is the intensity along the central axis. Then the FWHM is defined as the width at the half maximum of the curve. As shown in Supplementary Fig. 11c, two points can be resolved from the measurement if the distance between them is larger than the FWHM. Therefore, the lateral resolution Δx of our system is given by:

$$\Delta x = \int \varphi(y) dy,$$

$$\varphi(y) = \begin{cases} 1, & \bar{\phi}(x_{\frac{i}{2}}, y) > \frac{1}{2} max\left(\bar{\phi}(x_{\frac{i}{2}}, y)\right), \\ 0, & others, \end{cases} \tag{11}$$

where i is the number of vertical pixels of the diffused spot shown in the subfigures of Supplementary Fig. 11b-c. Supplementary Fig. 11d illustrates that the resolution of the system calculated by Supplementary Equation (11) is an increasing function of the depth.

To verify the derived theoretical resolution of our system, we perform experiments based on the measurements of a resolution chart. There are four groups of stripe patterns with centers separated by 5cm, 4cm, 3cm, and 2cm, as shown in Supplementary Fig. 12a. Supplementary Fig. 12b-d shows the reconstructions of the chart which is embedded in the polyethylene foam at the depths of 3cm, 4cm, and 5cm, respectively. Correspondingly, the total thicknesses of the foam are 10cm, 11cm, and 12cm. The laser scans a grid of 64 × 64 points on the front surface of the media over a 45cm × 45cm square area so that the scanning interval is 0.7cm which is smaller than the theoretical resolution at all depths used in our experiments. According to Supplementary Fig. 11d, the theoretical resolutions at the depths of 3cm, 4cm, 5cm are 3.3cm, 4.2cm, and 5.0cm, respectively. As can be seen from Supplementary Fig. 12b, the patterns separated by 3cm are just exceed the predicted resolution at a depth of 3cm, while the groups with 4cm and 5cm intervals are clearly resolvable. For the result at a depth of 4cm shown in Supplementary Fig. 12c, the groups separated by 5cm are clearly discernible, and the groups with 4cm intervals are also resolvable but a little fuzzy because this interval is just at the boundary of the theoretical resolution. Supplementary Fig. 12d shows the result at a depth of 5cm, the patterns separated by 5cm are resolvable as predicted by the theoretical values. The comparisons of theoretical and measured resolutions at different depths are summarized in the table below Supplementary Fig. 12, which presents high consistency between the experimental results and the derived theoretical resolutions.

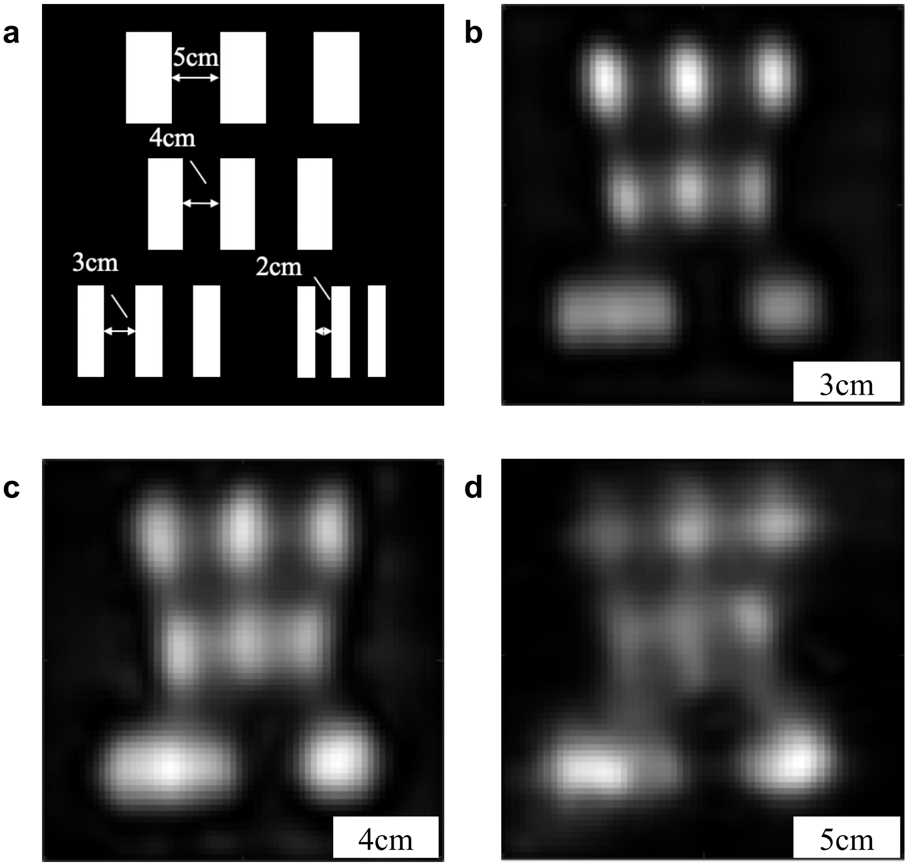

Depth (cm)	3	4	5
Theoretical Resolution (cm)	3.3	4.2	5.0
Measured Resolution (cm)	4.0	4.0	5.0

Supplementary Fig. 12. Reconstruction of the resolution chart to evaluate the system resolution in the polyethylene foam scattering scenario. a Resolution chart used in this section to evaluate the lateral resolution. There are four groups of stripe patterns that are separated by 5cm, 4cm, 3cm, and 2cm from the center of one stripe to another. **b-d** Reconstructions of the resolution chart at different depths: 3cm (**b**), 4cm (**c**) and 5cm (**d**). The comparisons of theoretical and measured resolutions at different depths are summarized in the table below.

Supplementary Note 5: Reconstructions of objects with different albedo surfaces

In this section, we show the performance of our algorithm to reconstruct objects with different albedo surfaces. The difference in properties of the target and the scattering media directly leads to the difference of the photons returned by the targets and the scattering media, which further affects the reconstruction performance. We have demonstrated the ability of our algorithm in reconstructing Lambertian objects in the experiments above. It is extremely challenging since the Lambertian surfaces diffuse the photons isotropically so that the photons returned by the object are highly coupled with those returned by the scattering media, as shown in Supplementary Fig. 10. While, the retroreflective surfaces on the non-Lambertian objects can considerably increase the direct-reflected signal photons with a much larger difference from the background scattered ones, which aids in the reconstruction. Therefore, many works reconstruct the retroreflective objects. Here, we also evaluate the performance of our algorithm on such objects to demonstrate its robustness.

Two-depth letters 'LU' and 'ST' are tested in the polyethylene foam scattering scenario in this experiment. As shown in Supplementary Table 4, the same letters are compared in three groups: both letters are Lambertian; one letter is Lambertian and the other is retroreflective; and the two letters are retroreflective. The depths of 'L' and 'U' are 3cm and 5cm, and the total thickness of the foam is 12cm. The depth of 'S' and 'T' are 4cm and 6cm, and the total thickness of the foam is 13cm. As a comparison, the results of the time gating algorithm are also given in Supplementary Table 4. It can be found that retroreflective surface allows for the better time-gated results of the closer objects such as 'U', while the time gating still does not work for the farther objects. In contrast, the proposed algorithm provides legible results in all three cases, which shows the robustness of our algorithm for reconstructing objects with different albedo surfaces. Additionally, we have shown that our algorithm can reconstruct the retroreflective object at the one-way optical depth of 28.5 TMFPs in Supplementary Table 3.

Reference	Captured Data	Time gating	Ours

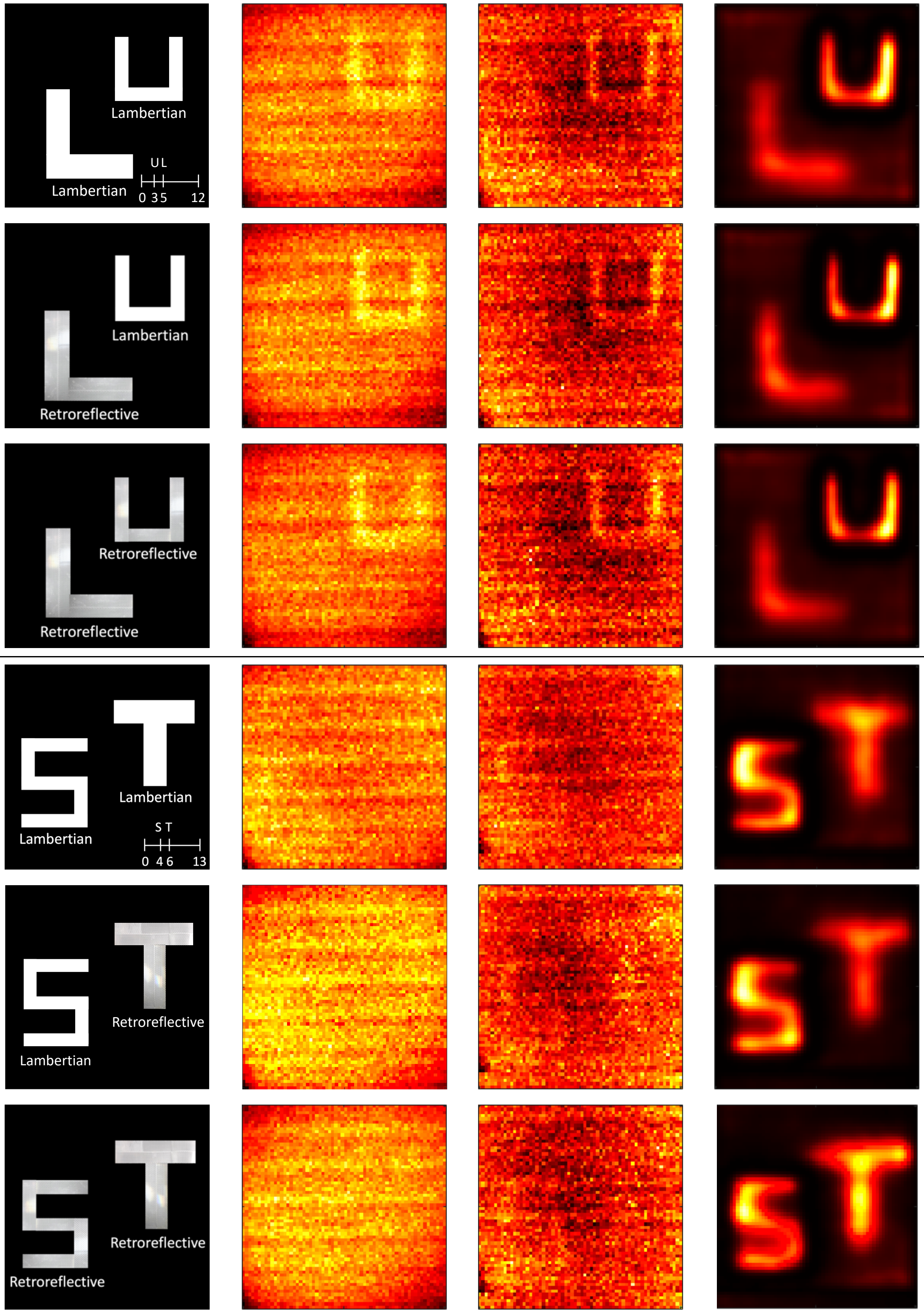

Supplementary Table 4. Reconstructions of objects with different albedo surfaces. Each set of two-depth letters is compared in three groups: both letters are Lambertian; one letter is Lambertian and the other is retroreflective; and the two letters are retroreflective.

Supplementary Note 6: Reconstructions of objects under ambient illumination

Different from the experiments above that are conducted in the dark environment, we demonstrate the ability of our algorithm under ambient illumination in this section. As shown in Supplementary Fig. 13a, 72W fluorescent lamps hanging 3m above the imaging scenario provide strong environmental lighting. Ambient illumination directly results in the increase of the background photons as the red curve shown in Supplementary Fig.13b, which makes the reconstruction more challenging. We compare the reconstructions of the two-depth letters 'ST' with different albedo surfaces under the ambient illumination, as shown in Supplementary Table 5. The depths of the 'S' and 'T' are 4cm and 6cm, respectively. The total thickness of the polyethylene foam is 13cm.

The change of illumination mode corresponds to the change of the source term on the right side of Eq. (1) in the main text. In our derivation, the source term is implicitly contained in two boundary conditions of the scattered field, $\Phi'(k_x, k_y, k_z)$ and $\bar{\Phi}(k_x, k_y, f)$ in Eq. (4) and Eq. (6) in the main text. Since we use a boundary migration model of the scattered field to tackle the scattering problem rather than directly calculate the analytical solution of Eq. (1), the specific form of the source term is not important. As can be seen from Supplementary Table 5, our algorithm under ambient illumination performs well as it does in the dark environment. Additionally, the reconstructions of different albedo surfaces show the robustness of our method for the object surface characteristics. Both of these indicate the potential of our algorithm in practical applications.

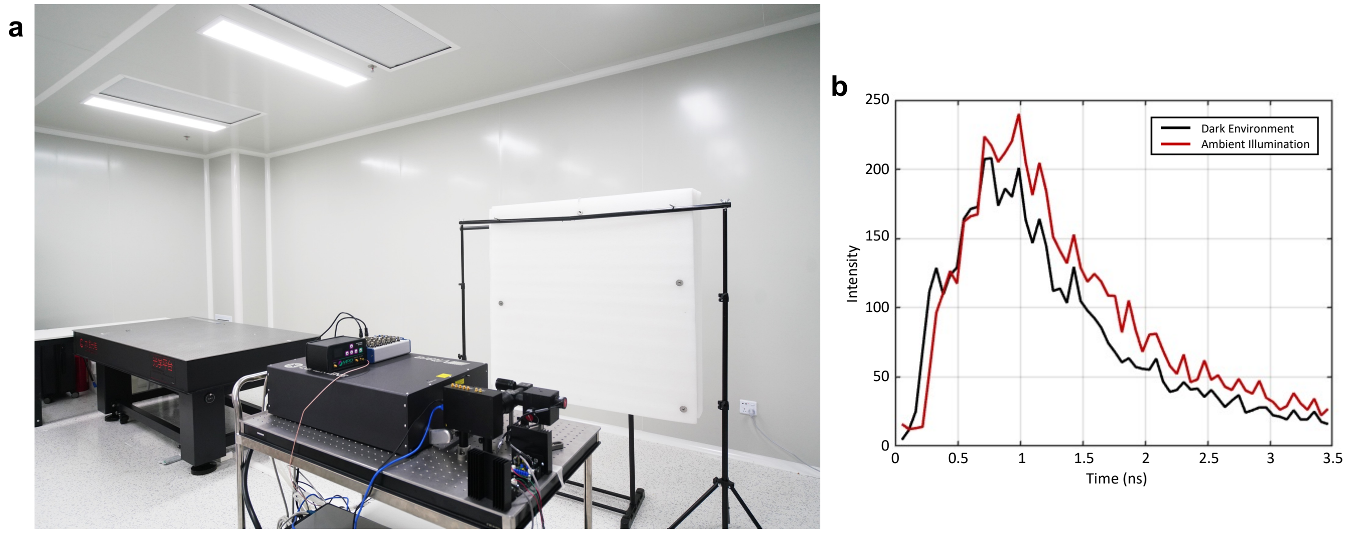

Supplementary Fig. 13. Experimental environment under ambient illumination. a 72W fluorescent lamps hanging 3m above the imaging scenario provide strong environmental lighting. **b** The black and red curves are the temporal responses measured under the dark environment and ambient illumination. It can be found that ambient illumination directly results in the increase of the background photons.

Reference	Dark Environment	Ambient Illumination
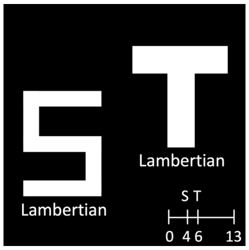	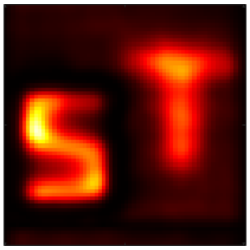	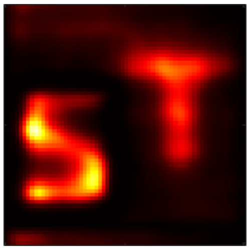
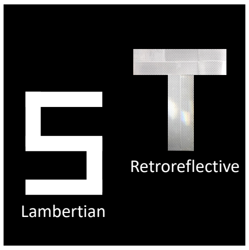	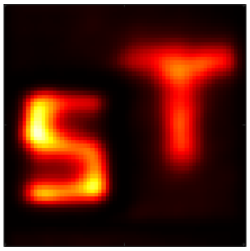	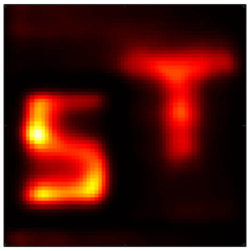
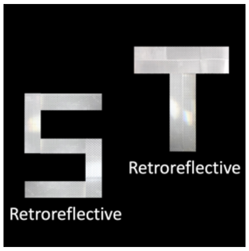	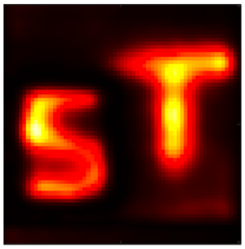	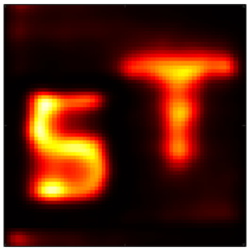

Supplementary Table 5. Reconstructions of objects with different albedo surfaces under the dark environment and ambient illumination. The experiments are conducted under the dark environment and ambient illumination. Two-depth letters 'ST' are compared in three groups: both letters are Lambertian; one letter is Lambertian and the other is retroreflective; and the two letters are retroreflective. The results show that the proposed algorithm under ambient illumination performs well as it does in the dark environment.

Supplementary Note 7: Comparison of relevant reconstruction methods

In this section, we compare our algorithm with the methods *f-k*[14] and CDT[15] which are also based on the idea of boundary value migration and are proposed in seeing around the corner and seeing through the scattering media, respectively. Both imaging scenarios are shown in Supplementary Fig. 14a-b. The difference among these three methods in principle is that *f-k* is based on the wave equation which only considers the propagation of light in free space, CDT models the scattering effect as a blur kernel acting on the free-space propagation, while our algorithm accurately models the light in scattering media based on the diffusion equation.

The difference is also reflected in the temporal response, as shown in Supplementary Fig. 14. It can be found that the temporal responses captured in the NLoS scenario are the same as those in the ideal single scattering scenario as shown in Fig. 1a in the main text and carry the shape information of the object in the discontinuities of the transient measurements. According to Fermat's principle[15], the types of transient discontinuities in temporal response, including local minimum, local maximum, or saddle point, correspond to the object's surface shape: convex, concave, or saddle, respectively. In this case, the red and green discontinuities of the temporal response in Supplementary Fig. 14 indicate that the vase surface shape is composed of a convex body and a saddle neck. With the increase in the scattering strength and the scattering region, the signal photons that carry the target information are drastically attenuated, as shown in Supplemental Fig. 14b or even almost disappeared in the volumetric scattering scenarios, as shown in Supplemental Fig. 14c. The temporal responses indicate that *f-k* and CDT both rely on the signal photons retained by light propagation in free space.

The reconstruction performance of the time gating, *f-k*, CDT and our algorithm are compared in the volumetric scattering scenario. First, we test the single Lambertian letter 'T' at different depths in the polyethylene foam, as shown in Supplementary Table 6. The temporal responses measured in such cases (Fig. 3b in the main text) show

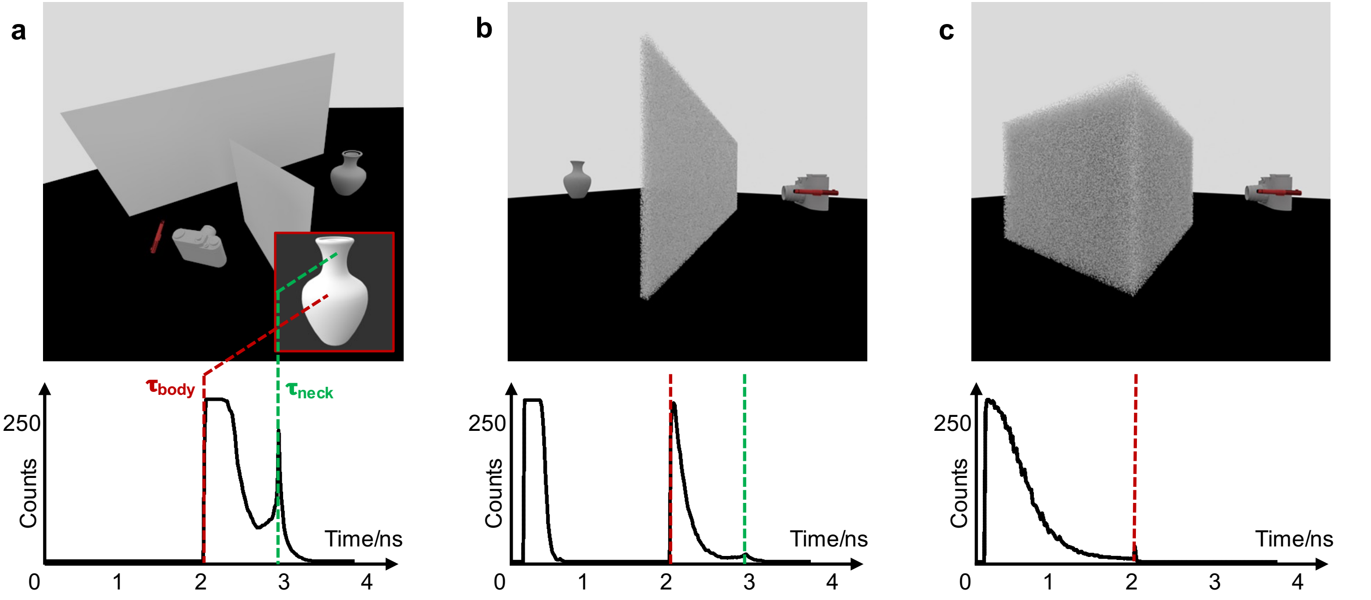

Supplementary Fig. 14. Illustration of different imaging scenarios. a seeing around the corner; **b** seeing through the scattering media; **c** seeing within the volumetric scattering media. The bottom row shows the temporal responses measured under different imaging scenarios. The red and green discontinuities in the transient measurements indicate that the vase surface shape is composed of a convex body and a saddle neck.

that it is difficult to directly decouple the signal photons from the measurements. Therefore, time gating, *f-k* and CDT cannot recover the object information from non-gated measurements. After cropping the measurement according to the object depth prior, time gating recovers the object at a depth of 2cm, *f-k* and CDT recover the object at 2cm and 4cm. Since the CDT models and inverts the scattering process, it performs better than *f-k*. However, time gating, *f-k* and CDT are all relied on the signal photons and thus they cannot handle the case where the signal photons are dramatically attenuated by the scattering media. In contrast, our algorithm models the propagation of light in the scattering media and computationally uses all the photons, which allows us to recover the object at the depth from 2cm to 8cm using non-gated measurements and provides the best performance.

Then we compare the reconstructions of multi-depth 2D objects and 3D objects embedded in the polyethylene foam in Supplementary Table 7. Since these objects have complex depth distributions, signal photons reflected from different depths will overlap in their temporal distribution. In this case, even using the measurements taken by combining multiple time-shift gates corresponding to different depths of the object, time gating, *f-k* and CDT provide almost no discernible information to identify the signatures of the object. Although these three algorithms reconstruct the letter ‘U’ at a relatively close depth of 3cm, the signal-to-noise ratio (SNR) of the results is low. In contrast, our method correctly recovers all the targets with high SNR and also provides the best qualitative agreement with the ground truth.

Supplementary Table 8 shows the comparison of these algorithms in reconstructing 3D objects with complex structure in the fog chamber. Since the fog is a typical forward scattering media where backscattered light has little influence on signal photons, the photons reflected by the object and those do not reach the object can be distinguished in the temporal response taking depth as a priori, as shown in Fig. 4b in the main text. In this case, time gating and *f-k* also provide partial discernible features of objects using cropped measurements. However, affected by the time variability of the fog, the temporal distribution of the signal photons is superimposed with dynamic noise so that time gating and *f-k* cannot reconstruct the exact shape of the object. Additionally, the CDT algorithm, which simply regards the scattering effect as a fuzzy kernel, is no longer suitable for the dynamic volumetric scattering media and thus fails to recover the objects embedded in the fog. In contrast, our algorithm successfully recovers all the objects, especially the details of objects such as the handle and lid of the ‘Teapot’ and the ear of the ‘Bunny’.

Exposure time per scan position, photon counts per histogram and runtime of the above experiments are summarized in Supplementary Table 9. The runtime is measured on a MacBook Pro (2.3GHz Intel Core i5) under MATLAB environment. Even though we do not crop the data to remove the photons directly returned by the scattering media, the number of photons per histogram used in our experiment is 1-2 orders smaller than that in the CDT experiment as described in the respective paper. The average exposure time per scan position in our experiment is around 15 times shorter than that in the CDT experiment. Additionally, the runtime of our algorithm is almost equal to that of *f-k* and around 3 times faster than that of CDT. The runtime and the requirement of data acquisition including photon counts and exposure time show the potential of our algorithm for real-time applications.

Depth	2cm 6.3 TMFPs	4cm 12.7 TMFPs	6cm 19.0 TMFPs	8cm 25.4 TMFPs
Reference	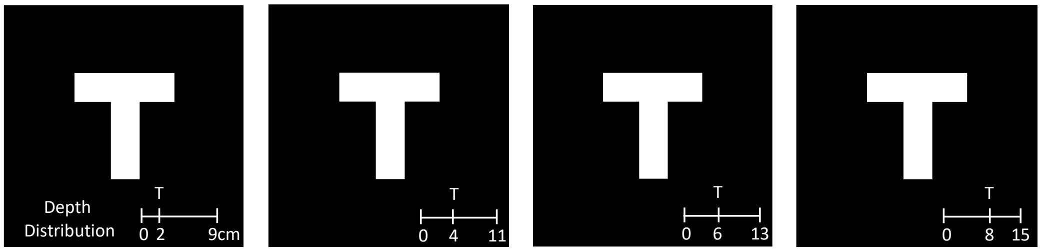			
Captured Data	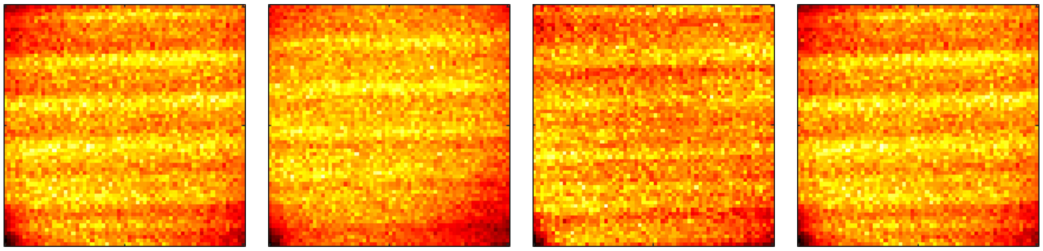			
Time gating	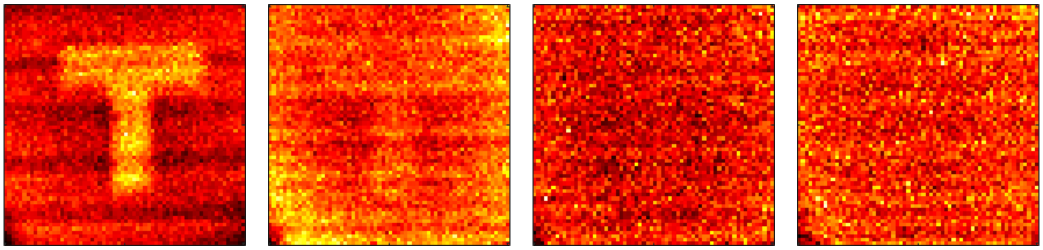			
f-k[14]	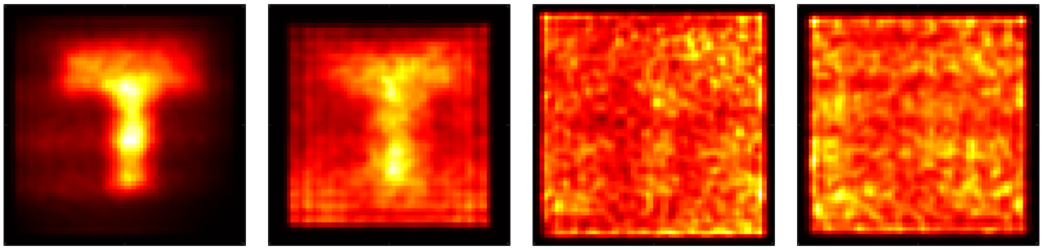			
CDT[16]	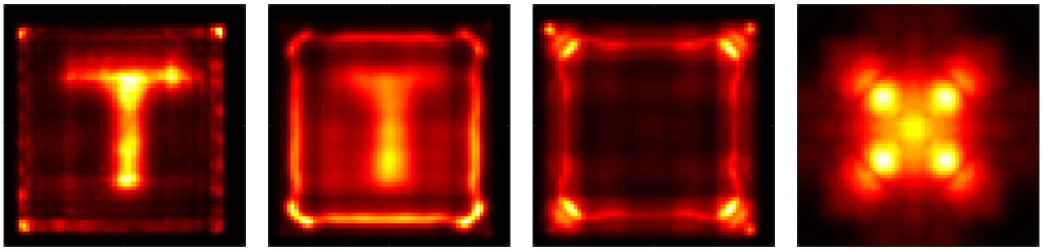			
Ours	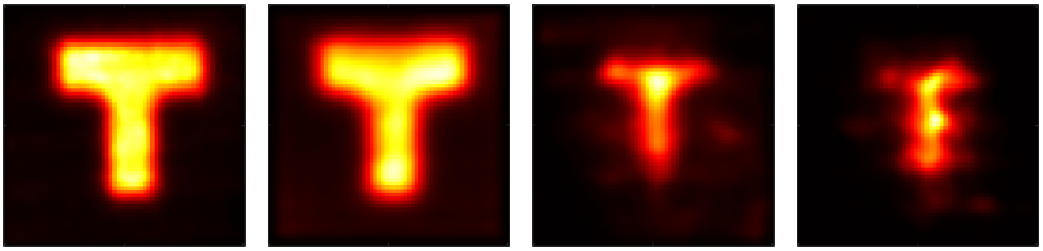			

Supplementary Table 6. Comparison of time gating, *f-k*, CDT and our algorithm for reconstructing single letter 'T' at different depths in the polyethylene foam. The total thicknesses of the foam and the object depths are marked in the lower right corner of the reference figures.

Depth	U-3cm L-5cm	S-4cm T-6cm	3-7cm	3-7cm
Reference	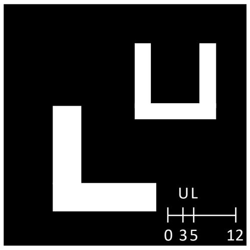	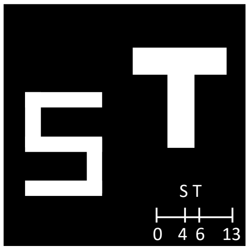	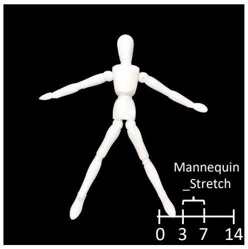	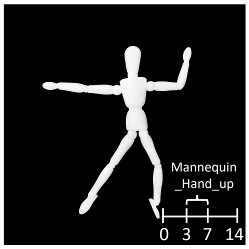
Captured Data	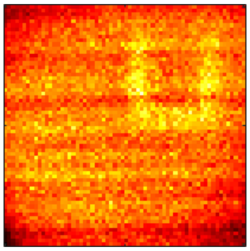	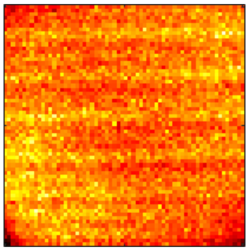	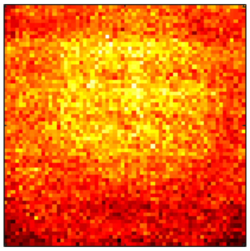	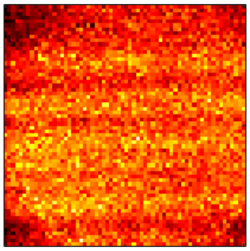
Time gating	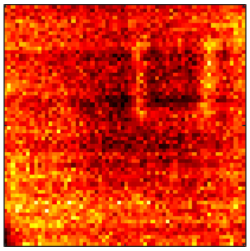	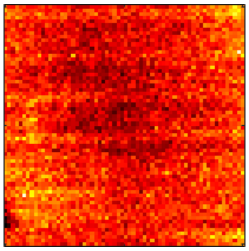	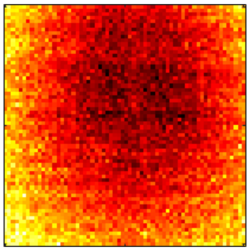	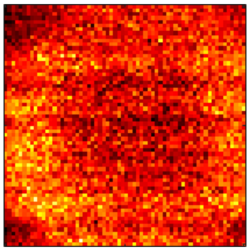
f-k[14]	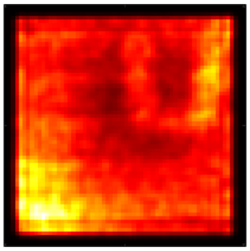	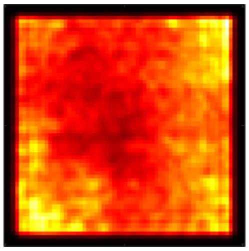	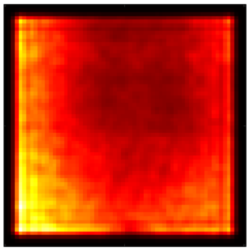	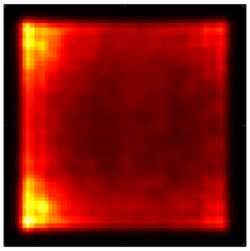
CDT[16]	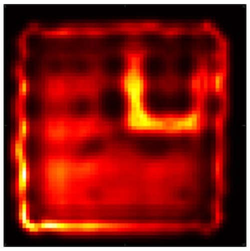	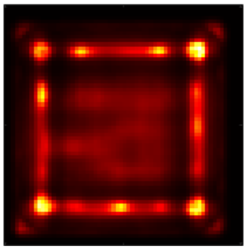	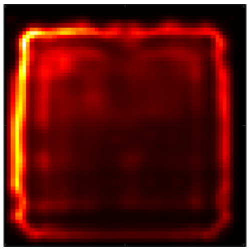	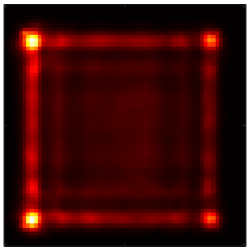
Ours	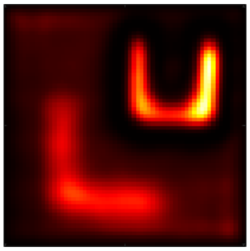	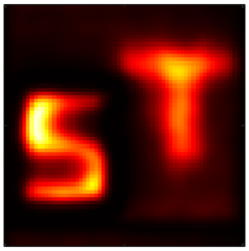	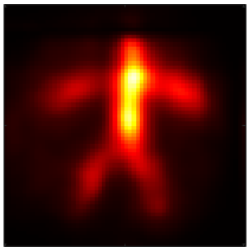	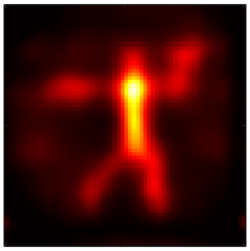

Supplementary Table 7. Comparison of time gating, *f-k*, CDT and our algorithm in reconstructing 2D objects with multiple discrete depths and 3D objects in the polyethylene foam. The total thicknesses of the foam and the object depths are marked in the lower right corner of the reference figures.

Depth	45-60cm	50-65cm	40-60cm
Reference	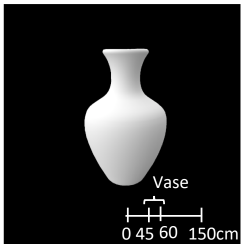	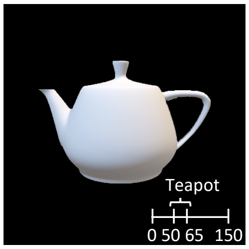	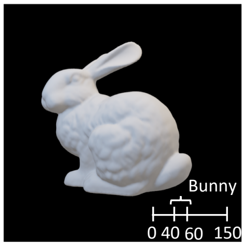
Captured Data	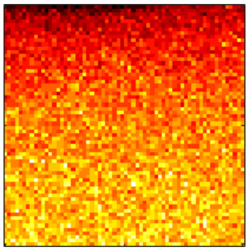	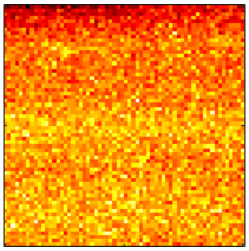	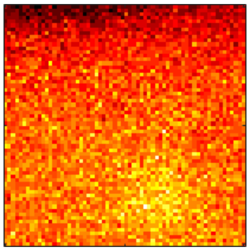
Time gating	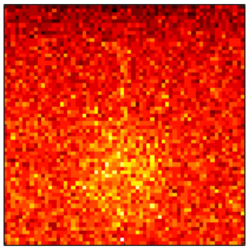	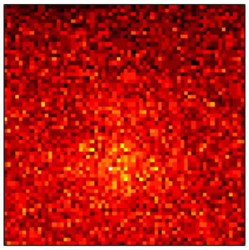	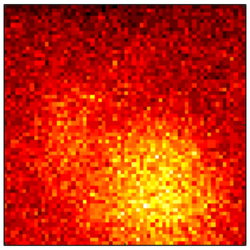
f-k[14]	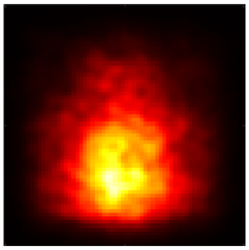	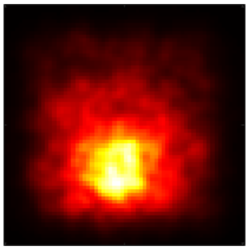	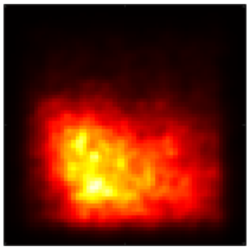
CDT[16]	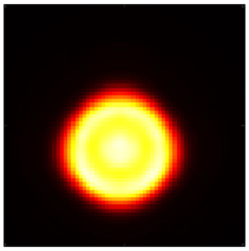	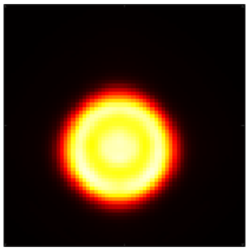	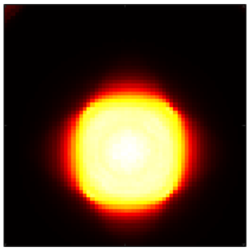
Ours	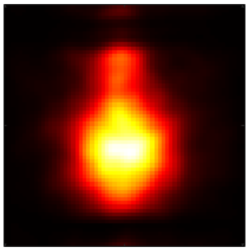	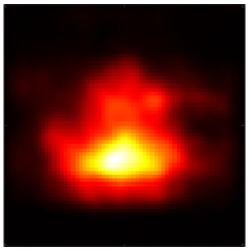	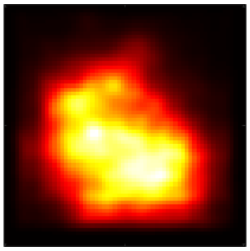

Supplementary Table 8. Comparison of time gating, *f-k*, CDT and our algorithm for reconstructing 3D objects with complex structure in the fog chamber. The total depth of the fog chamber and the object depth are marked in the lower right corner of the reference figures.

Experiment	Data name	Photon counts	Exposure time (ms)	Runtime (s)		
				f-*k*[14]	CDT[16]	proposed
Supplementary Table 6	‘T’ (2cm)	6100	60	0.490	2.741	0.529
	‘T’ (4cm)	7108	60	0.511	1.602	0.497
	‘T’ (6cm)	6474	60	0.482	1.577	0.493
	‘T’ (8cm)	6489	60	0.576	1.714	0.562
Supplementary Table 7	‘LU’	4975	60	0.512	1.638	0.505
	‘ST’	5418	60	0.527	1.351	0.494
	‘ Mannequin_Stretch’	2679	60	0.555	1.446	0.560
	‘Mannequin_Hand_up’	2692	60	0.473	1.709	0.479
Supplementary Table 8	‘Vase’ (fog)	263	20	0.381	1.799	0.364
	‘Teapot’	306	20	0.453	1.567	0.470
	‘Bunny’	324	20	0.476	1.792	0.480
Main text Fig. 5	‘Vase’ (foam)	2867	60	0.497	1.406	0.504

Supplementary Table 9. Exposure time, photon counts, and runtime for all experiments. Exposure time per scan point, photon counts per histogram, and runtime of the different algorithms for the above measurements are summarized in this table.

Supplementary Note 8: Discussion of Depth Reconstruction

In this section, we discuss the ability of our algorithm in depth reconstruction. During our derivation, the depth information is included in the temporal boundary condition of the scattered field $\phi(x, y, z, t = 0)$, and transformed into the frequency domain as $\Phi'(k_x, k_y, k_z)$. To make it computationally feasible to establish the transformation relationship between the temporal and spatial boundary condition of the scattered field. The dispersion relation is used to transform the wave vector of depth k_z to frequency f, so that $\Phi'(k_x, k_y, k_z)$ is weighted and interpolated to be $\overline{\Phi}(k_x, k_y, f)$. Then the spatial boundary condition $\phi(x, y, z = 0, t)$ is obtained by applying the inverse Fourier transform over k_x, k_y and a numerical transformation over f using Eq. (6) in the main text. In this case, the depth information of the object is reflected in the temporal information of the measurements. However, the transformation from $\phi(x, y, z = 0, t)$ to $\overline{\Phi}(k_x, k_y, f)$ over t is an ill-conditioned problem as shown in Eq. (8) in Methods. Specifically, the transfer matrix A between f and t is a sparse matrix. Inverting sparse matrices is an ill-conditioned problem. Methods such as the Quasi-minimal residual method, Preconditioned Conjugate Gradient (PCG), or Generalized Minimal Residual (GMRES) can reduce the residuals of sparse matrix inversion at some level, while solving the inverse problem still affects the accuracy of our algorithm in depth reconstruction.

We compare the depth reconstructions of time gating, *f-k*, CDT and our algorithm in Supplementary Table 10. The target is a bunny embedded in the fog chamber and the experimental condition is the same as that described in the main text. The front view, top view, and side view are given by the maximum projection of the 3D reconstructions in *x-y*, *x-z,* and *y-z* dimensions, respectively. The reconstructed depth information can be seen from the top and side view. The results captured without scattering media are used as the ground truth in this comparison. Since the time-gated result is obtained by directly cropping the time bins according to the object depth prior, it reflects the depth distribution of the object to some extent. Suffering from the facts that *f-k* does not consider the scattering effect, CDT does not accurately model the propagation in the volumetric scattering media, and our algorithm is affected by the error in solving the ill-conditioned problem, these three methods can not accurately recover the depth of the object. Fortunately, the rough depth of the object can be directly estimated from the temporal response in the fog since the backscattered light has little effect on the objects, as shown in Fig. 4b in the main text. This estimated depth range can be used as a criterion to calibrate the original depth reconstructed by *f-k*, CDT and our algorithm. In this case, the top view and side view of the final reconstructions using these three methods can give the rough depth of the object as shown in the bottom three rows in Supplementary Table 10. Additionally, the depth reconstruction performance of *f-k* is better than that of CDT and our algorithm because it recovers not only the rough depth range but also the details of the depth distribution of the object. Our algorithm loses part of depth information in the process of inverting the ill-conditioned problem and thus fails to reconstruct the accurate details of the depth distribution of objects. We will explore other relative algorithms aiming at solving the inversion of the large sparse matrix to help accurately recover the depth information in the future.

Depth	Front view (*x*-*y*)	Top view (*x*-*z*)	Side view (*y*-*z*)
No Scattering	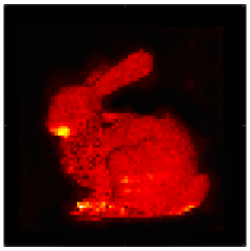	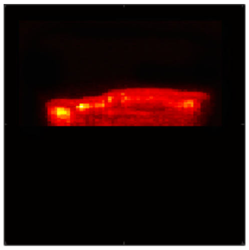	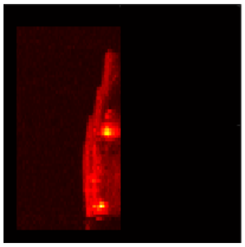
Time gating	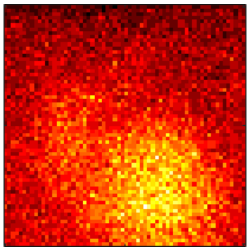	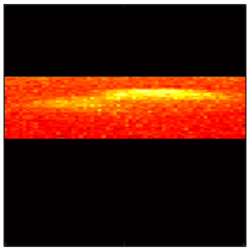	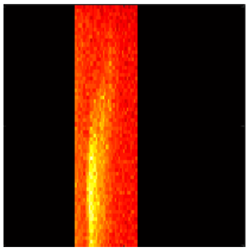
f-*k*[14]	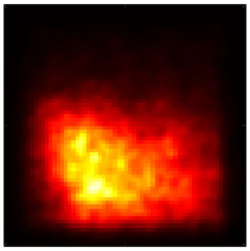	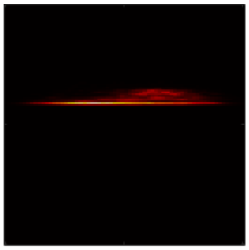	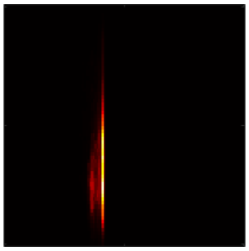
CDT[16]	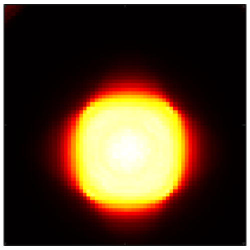	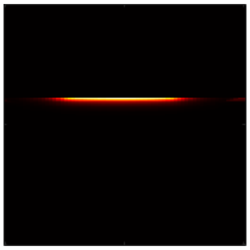	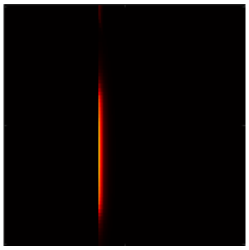
Ours	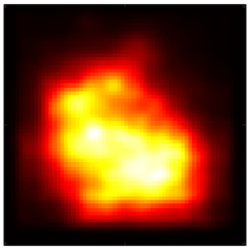	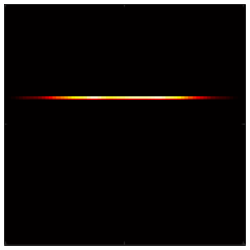	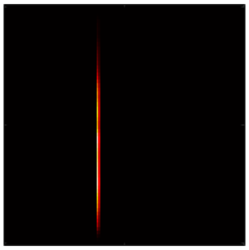

Supplementary Table 10. Comparison of time gating, *f-k*, CDT and our algorithm in depth reconstruction. The result captured without scattering media is used as the ground truth. The front view, top view, and side view are given by the maximum projection of the 3D reconstructions in *x*-*y*, *x*-*z,* and *y*-*z* dimensions, respectively.

Supplementary References

1. Metari S, Deschenes F, A new convolution kernel for atmospheric point spread function applied to computer vision, in *Proceedings of the 2007 IEEE International Conference on Computer Vision*, 474-481 (2007).
2. Colombi J, Louedec K. Monte Carlo simulation of light scattering in the atmosphere and effect of atmospheric aerosols on the point spread function. *Journal of the Optical Society of America a-Optics Image Science and Vision* **30**, 2244-2252 (2013).
3. Grabner M, Kvicera V. Multiple Scattering in Rain and Fog on Free-Space Optical Links. *Journal of Lightwave Technology* **32**, 513-520 (2014).
4. Patterson MS, Chance B, Wilson BC. Time resolved reflectance and transmittance for the noninvasive measurement of tissue optical properties. *Applied Optics* **28**, 2331-2336 (1989).
5. Contini D, Martelli F, Zaccanti G. Photon migration through a turbid slab described by a model based on diffusion approximation .2. Theory. *Applied Optics* **36**, 4587-4599 (1997).
6. Farrell TJ, Patterson MS, Wilson B. A diffusion theory model of spatially resolved, steady-state diffuse reflectance for the noninvasive determination of tissue optical properties in vivo. *Medical Physics* **19**, 879-888 (1992).
7. Zhu JX, Pine DJ, Weitz DA. Internal reflection of diffusive light in random media. *Physical Review A* **44**, 3948-3959 (1991).
8. Yoo KM, Feng L, Alfano RR. When does the diffusion approximation fail to describe photon transport in random media? *Physical Review Letters* **64**, 2647 (1990).
9. Gardiner CW. Diffusive traversal time of a one-dimensional medium. *Journal of Statistical Physics* **49**, 827-828 (1987).
10. Landauer R, Buttiker M. Diffusive traversal time: Effective area in magnetically induced interference. *Physical Review B Condensed Matter* **36**, 6255 (1987).
11. Morales-Cruzado B, Sarmiento-Gómez E, Camacho-López S, Pérez-Gutiérrez F. Nanosecond laser pulse propagating through turbid media: a numerical analysis. *Revista mexicana de física* **63**, 89-96 (2017).
12. Papazoglou TG, Liu WQ, Vasiliou A, Grassmel R, Papagiannakis E, Fotakis C. Limitations of diffusion approximation in describing femtosecond laser transillumination of highly scattering media of biological significance. *Applied Physics Letters* **67**, 3712-3714 (1995).
13. Seo I, Hayakawa CK, Venugopalan V. Radiative transport in the delta-P-1 approximation for semi-infinite turbid media. *Medical Physics* **35**, 681-693 (2008).
14. Lindell DB, Wetzstein G, O'Toole M. Wave-based non-line-of-sight imaging using fast f-k migration. *ACM Transactions on Graphics* **38**, 116 (2019).
15. Xin S*, et al.*, A Theory of Fermat Paths for Non-Line-of-Sight Shape Reconstruction, in *Proceedings of the 2019 IEEE Conference on Computer Vision and Pattern Recognition*, 6793-6802 (2019).
16. Lindell DB, Wetzstein G. Three-dimensional imaging through scattering media based on confocal diffuse tomography. *Nature Communications* **11**, 4517 (2020).
